# Genetic Diversity and Phylogenetic Differentiation of Southwestern Chinese Han: a comprehensive and comparative analysis on 21 non-CODIS STRs

**DOI:** 10.1038/s41598-017-13190-w

**Published:** 2017-10-23

**Authors:** Guanglin He, Zheng Wang, Mengge Wang, Yiping Hou

**Affiliations:** Institute of Forensic Medicine, West China School of Basic Science and Forensic Medicine, Sichuan University, Chengdu, 610041 China

## Abstract

Short tandem repeats (STRs), with high polymorphism and complex evolution information, play a significant role in genetic association studies like population genetics, molecular anthropology and human forensics. However, human genetic diversity has only been partially sampled and available for Southwest Chinese Han population, as well as the genetic architecture of this population remains uncharacterized. In this work, 368 unrelated Han individuals from Sichuan province were firstly genotyped with 21 non-CODIS autosomal STRs, and phylogenetic relationships along administrative (Han Chinese from different regions) and ethnic divisions (minority ethnic groups) were subsequently investigated. The CMP and CPE were 6.2796 × 10^−20^ and 0.9999999, respectively. Analysis of molecular variance (AMOVA), principal component analysis (PCA), multidimensional scaling plots (MDS) and phylogenetic analysis consistently demonstrated that the Southwest Han population had a close genetic relationship with the geographically close population (Hunan Han) and kept a distant genetic relationship with some ethnic groups, most prominently for Gansu Yugu and Fujian She. Furthermore, no significant genetic distinction between the Northern Han and Southern Han was observed. Aforementioned results suggested that these 21 STRs are highly polymorphic and informative, which are suitable for human identification and population genetics.

## Introduction

Short tandem repeats (STRs), scattered throughout the genome with high heterozygosity and evolutionary information, are attractive to genetic applications like human forensics, anthropological and population genetics studies^1–5^. In the past decades, DNA profiling based on a set of core STRs was employed in national DNA database construction, forensic personal identification and parentage testing in the forensic community^6^. 13 autosome STRs were initially chosen to constitute the Combined DNA Index System (CODIS) by the Federal Bureau of Investigation Laboratory^2^, while a parallel process occurred in European where the European Standard Set (ESS)^7^ based on 12 autosome STRs. In order to increase the power of discrimination (PD), to minimize adventitious matches and to increase international compatibility, additional 7 and 4 STRs were respectively added to the CODIS and ESS recently^7,8^. China, the world’s most populous country, has the largest national law enforcement DNA database and mainly STR profiles were generated based on the CODIS core loci^9–12^. Currently, serval commercial PCR amplification kits on basis of CODIS loci, such as the Huaxia Platinum System (Thermo Fisher Scientific, MA, USA), AGCU EX22 (AGCU ScienTech Incorporation), PowerPlex Fusion Systems (Promega, WI, USA) and others^13–19^, are widely used in China. However, as the volume of data under management seems to be increasing even faster, more novel STR loci with high genetic polymorphisms are required to minimize the incidence of adventitious matches.

The AGCU 21 + 1 System (AGCU ScienTech Incorporation) was specifically developed for forensic application in Chinese populations^20^. This system is a 22-locus, five-dye, multiplex that allows co-amplification and fluorescent detection of the 21 non-CODIS STRs (D10S1248, D10S1435, D11S4463, D12ATA63, D14S1434, D17S1301, D18S853, D19S433, D1GATA113, D1S1627, D1S1677, D20S482, D22S1045, D2S1776, D2S441, D3S4529, D4S2408, D5S2500, D6S1017, D6S474, and D9S1122) as well as Amelogenin for gender determination. In addition to significantly improving forensic efficiency, these 21 STRs hold the potential to investigate the recent evolutionary events and population substructure within China^21–45^.

Recently, human genetic diversity has only been partially sampled and was available for 56 Chinese officially recognized populations. To the best of our knowledge, the information of genetic architecture and population genetic relationship of Sichuan Han population (Southwestern Chinese Han) remains limited^46^. Sichuan province, the provincial capital Chengdu, is located in southwest China, occupying most of the Sichuan Basin between the Himalayas on the west, the Daba Mountains in the north, and the Yungui Plateau to the east. Sichuan is the fifth largest Chinese province, with population accounting for 6% of the total population. The majority of the province’s population is Han Chinese, who are found scattered throughout the administrative region with the exception of the far western areas. In this study, we firstly obtained the genetic polymorphism data of 21 non-CODIS STRs of 368 Han Chinese individuals to provide forensic reference databases in the Southwest Han population. Subsequently, genetic population data of these 21 STRs (9444 individuals) were collected from public databases and reported literature^21–45^ to investigate Chinese population substructure using analysis of molecular variance (AMOVA), principal component analysis (PCA), multidimensional scaling plots (MDS) and phylogenetic analysis.

## Results and Discussion

Covering approximately 9.6 million square kilometers, China is the world’s second-largest state by land area, consisting of 22 provinces, four direct-controlled municipalities, five autonomous regions and two mostly self-governing special administrative regions. China is the world’s most populous country, with a population of over 1.4 billion. As the biggest ethnic group, the Han population constitutes about 92% of the total population in China. Han Chinese are the majority in every municipality, province, autonomous region and the special administrative regions except for the autonomous regions of Tibet and Xinjiang. In addition to the Han Chinese group, 55 ethnic minority groups within China have been officially recognized, which vary in culture and social customs. In this work, genetic polymorphisms of 21 non-CODIS STRs in Southwest Chinese Han population (Sichuan province) were firstly obtained. Subsequently, all previously reported 21 non-CODIS STRs data in Chinese populations^21–45^ were integrated to explore the genetic diversity and the population substructures. Finally, a total of 26 populations consisted of 9444 individuals were included in population relationship comparisons (Supplementary Table S1).

### Genetic parameters of the 21 non-CODIS STRs

A total of 368 unrelated Chinese Han individuals residing in Sichuan Province were genotyped using a multiplex assay amplifying 21 non-CODIS autosomal STR loci (AGCU 21 + 1 System). Forensic parameters including observed heterozygosity (H_o_), expected heterozygosity (H_e_), power of discrimination (PD), power of exclusion (PE), polymorphism information content (PIC) and typical paternity index (TPI) for each locus are shown in Table 1 and detailed genotypes are listed in Supplementary Table S2. No significant deviations from Hardy-Weinberg equilibrium were observed for any of the 21 non-CODIS autosomal STR loci after Bonferroni correction (p > 0.002380), and no evidence of linkage inheritance of all pairwise loci after the correction for multiple tests (Supplementary Table S3).

**Table 1:** The forensic parameters for 21 STRs in Southwest Han population samples (n = 368).

Allele	H_O_	H_E_	PD	PE	PIC	TPI	p-value
D10S1248	0.7337	0.7499	0.8936	0.4823	0.7096	1.8776	0.4722
D10S1435	0.7799	0.7475	0.8929	0.5623	0.7064	2.2716	0.1531
D11S4463	0.8016	0.7660	0.8984	0.6021	0.7269	2.5205	0.1061
D12ATA63	0.7092	0.7332	0.8834	0.4427	0.6862	1.7196	0.2983
D14S1434	0.7391	0.7098	0.8619	0.4914	0.6645	1.9167	0.2149
D17S1301	0.7147	0.7088	0.8761	0.4513	0.6678	1.7524	0.8031
D18S853	0.6902	0.7239	0.8853	0.4133	0.6789	1.6140	0.1486
D19S433	0.8179	0.8082	0.9372	0.6327	0.7825	2.7463	0.6358
D1GATA113	0.6332	0.6450	0.8132	0.3326	0.5794	1.3630	0.6340
D1S1627	0.5842	0.6095	0.7890	0.2724	0.5516	1.2026	0.3200
D1S1677	0.6467	0.6695	0.8358	0.3508	0.6124	1.4154	0.3533
D20S482	0.7364	0.7277	0.8755	0.4868	0.6819	1.8969	0.7061
D22S1045	0.7962	0.7585	0.8910	0.5920	0.7148	2.4533	0.0914
D2S1776	0.7690	0.7445	0.8813	0.5429	0.7041	2.1647	0.2805
D2S441	0.7473	0.7819	0.9176	0.5051	0.7477	1.9785	0.1074
D3S4529	0.7582	0.7543	0.8952	0.5238	0.7112	2.0674	0.8643
D4S2408	0.7201	0.7414	0.8887	0.4601	0.6955	1.7864	0.3509
D5S2500	0.7283	0.7013	0.8481	0.4733	0.6467	1.8400	0.2579
D6S1017	0.7473	0.7258	0.8755	0.5051	0.6818	1.9785	0.3565
D6S474	0.7283	0.7017	0.8496	0.4733	0.6477	1.8400	0.2652
D9S1122	0.6766	0.7109	0.8675	0.3930	0.6606	1.5462	0.1474

A total of 177 alleles were observed with the corresponding allelic frequencies varied from 0.0014 to 0.5476. D2S441 was detected with the 13 alleles at the maximum, while D1GATA113 was only detected with 5 alleles in Sichuan Han population (Supplementary Table S4). As shown in Table 1, the H_o_ and H_e_ ranged from 0.5842 to 0.8179 (0.7266 ± 0.0571), 0.6095 to 0.8082 (0.7247 ± 0.0449), respectively. The PIC and TPI spanned from 0.5516 (D1S1627) to 0.7825 (D19S433), 1.2026 (D1S1627) to 2.7463 (D19S433), respectively. The locus with the largest PD was D19S433 (0.9327), and the combined match probability (CMP) value was 6.2796 × 10^−20^. The highest and lowest PE loci were D19S433 (0.6327) and D1S1627 (0.2724), respectively, and the combined power of exclusion (CPE) value was 0.9999999. Aforementioned results suggested that the 21 non-CODIS STRs are polymorphic and informative in Sichuan Han population. This study provided the first batch of genetic diversity information of 21 non-CODIS STRs in Southwest Chinese Han population and enriched the Chinese non-CODIS STRs reference databases.

### Population pairwise differences and principal component analysis

In order to illuminate the genetic relationships between the Sichuan Han population and previously reported populations, 12 Han Chinese populations residing in different geographic regions (total number is 6212), one Xinjiang Kazakh (n = 114), one Hainan Li (n = 504), one Inner Mongolia Mongolian (n = 523), one Inner Mongolia Russian (n = 114), one Qinghai Salar (n = 120), one Fujian She (n = 154), one Lhasa Tibetan (n = 104), one Hubei Tujia (n = 107), one Xinjiang Uyghur (n = 502), one Xinjiang Xibe (n = 226), one Yunnan Yi (n = 110), one Gansu Yugu (n = 180) and one Yunnan Bai (n = 106) were included in the population comparison study (Supplementary Table S1). The locus-by-Locus F_st_ and corresponding p values between the Sichuan Han and aforementioned reference populations are presented in Supplementary Table S5. Four STR loci (D3S4529, D12ATA63, D20S482 and D22S1045) in Gansu Yugu, four loci (D6S1017, D10S1248, D20S482 and D22S1045) in Fujian She, three loci (D6S1017, D12ATA63, D22S1045) in Lhasa Tibetan, two loci (D1S1677 and D4S2408) in Inner Mongolia Mongolia, two loci (D1S1627 and D12ATA63) in Hainan Li, two loci (D10S1435 and D22S1045) in Zhejiang Han, D22S1045 in Qinghai Salar, D22S1045 in Northern Han, D22S1045 in Yunnan Yi, D22S1045 Inner Mongolian Russian were observed significant difference (p < 0.002, after Bonferroni adjustment) with Sichuan Han using AMOVA.

**Figure 1:**
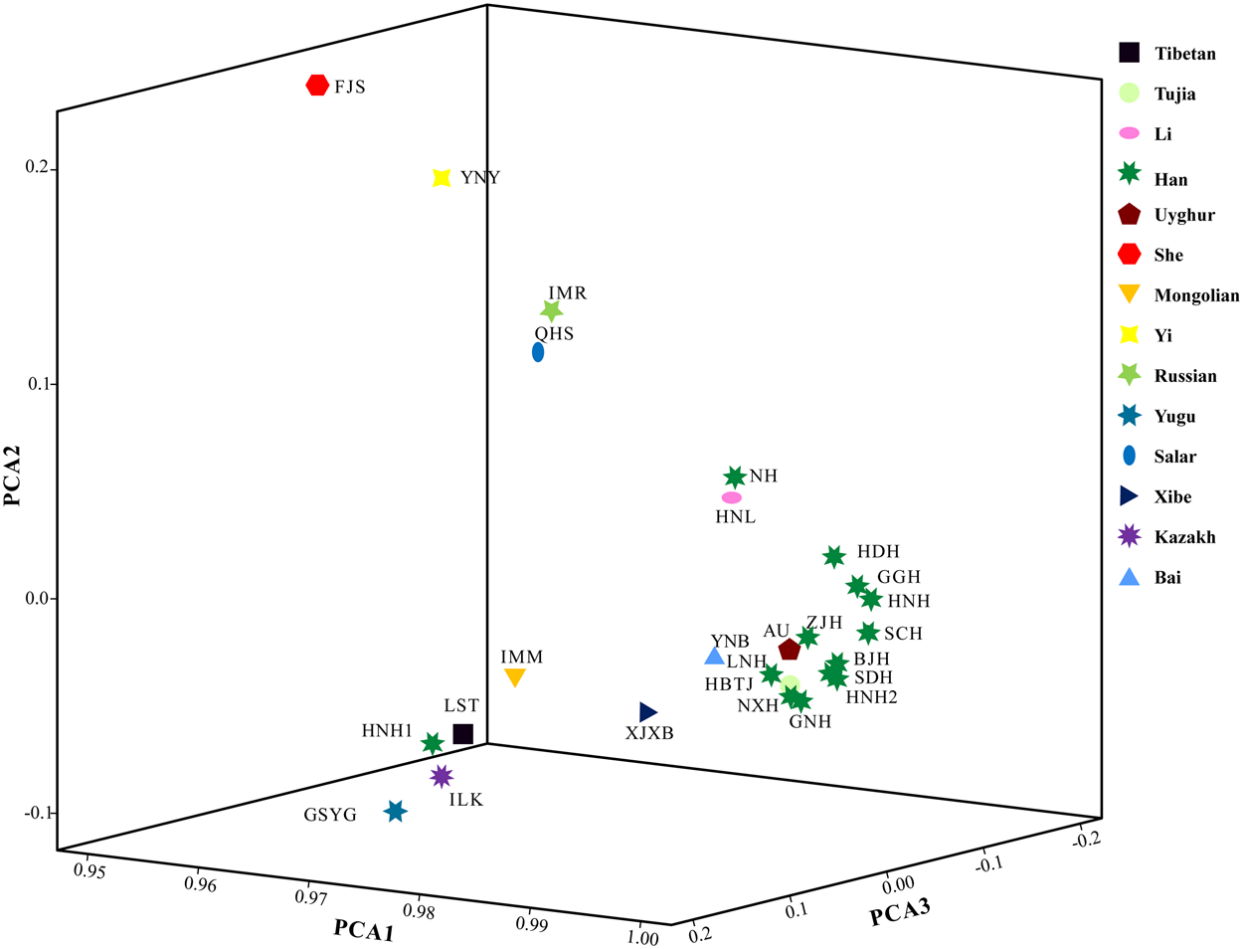
Principal component analysis plot structured based on allelic frequencies of 21 non-CODIS STRs in 26 populations.

PCA extracted several components as new variables by the dimension reduction method, which can be utilized to determine basic characteristic features for differentiation among groups. According to Fig. 1, the first principal component defined 96.826% of the total variance, and the second and third principal components accounted for 0.624% and 0.497% of the total variance, respectively. Han Chinese populations clustered together and distributed in the right lower part, whereas Chinese ethnic groups distributed separately. Specifically, Fujian She, Yunnan Yi, Inner Mongolian Russian, Qinghai Salar and Hainan Li distributed in the left upper part, and Gansu Yugu, Ili Kazakh, Lhasa Tibetan, Inner Mongolia Mongolian, Xinjiang Xibe distributed in left lower part.

### MDS analysis and phylogenetic structure

**Figure 2:**
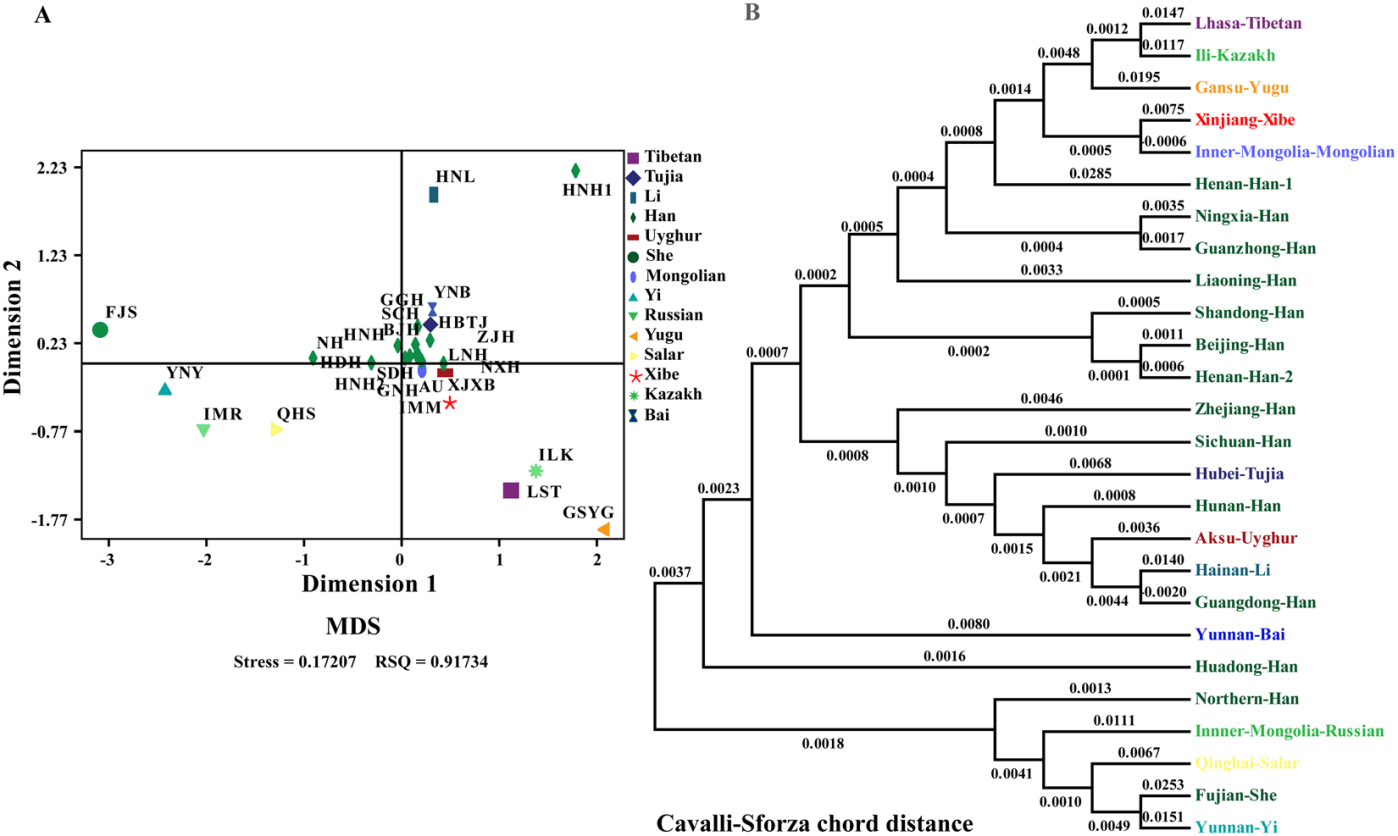
The MDS plot and neighbor-joining phylogenetic tree constructed based on the Cavalli-Sforza and Edward’s chord distances (D_CE_).

Three distinct genetic distances (Cavalli-Sforza Edward’s chord distance (D_CE_), Nei’s genetic distance and Reynolds genetic distance) were performed to explore the genetic differentiation. The D_CE_ genetic distances between the Sichuan Han and other 25 Chinese populations are listed in Supplementary Table S6. Among 25 reference populations from separate administrative divisions or groups, Fujian She is the most distantly related to the Sichuan Han (D_CE_ = 0.0090), followed by Gansu Yugu (D_CE_ = 0.0079), Yunnan Yi (D_CE_ = 0.0078), whereas Hunan Han, Shandong Han and Henan Han 2 are the closest with our investigated group (D_CE_ = 0.0011). The Multidimensional Scaling plots and a neighboring joining tree on the basis of the D_CE_ pairwise genetic distance matrix were used to infer evolutionary relationships. As shown in Fig. 2A, 9 out of 26 populations (Gansu Yugu, Lhasa Tibetan, Ili Kazakh, Qinghai Salar, Inner Mongolia Russian, Yunnan Yi, Fujian She and Hainan Li, and Henan Han 1) were isolated. Additional Chinese Han populations residing in different Chinese administrative divisions and ethnic groups (Yunnan Bai, Xinjiang Xibe, Inner Mongolia Mongolian, Hubei Tujia and Aksu Uyghur) were clustered. The lower clade and most upper sub-clade were consisted of ethnic groups, whereas Han Chinese populations were grouped together (Fig. 2B).

**Figure 3:**
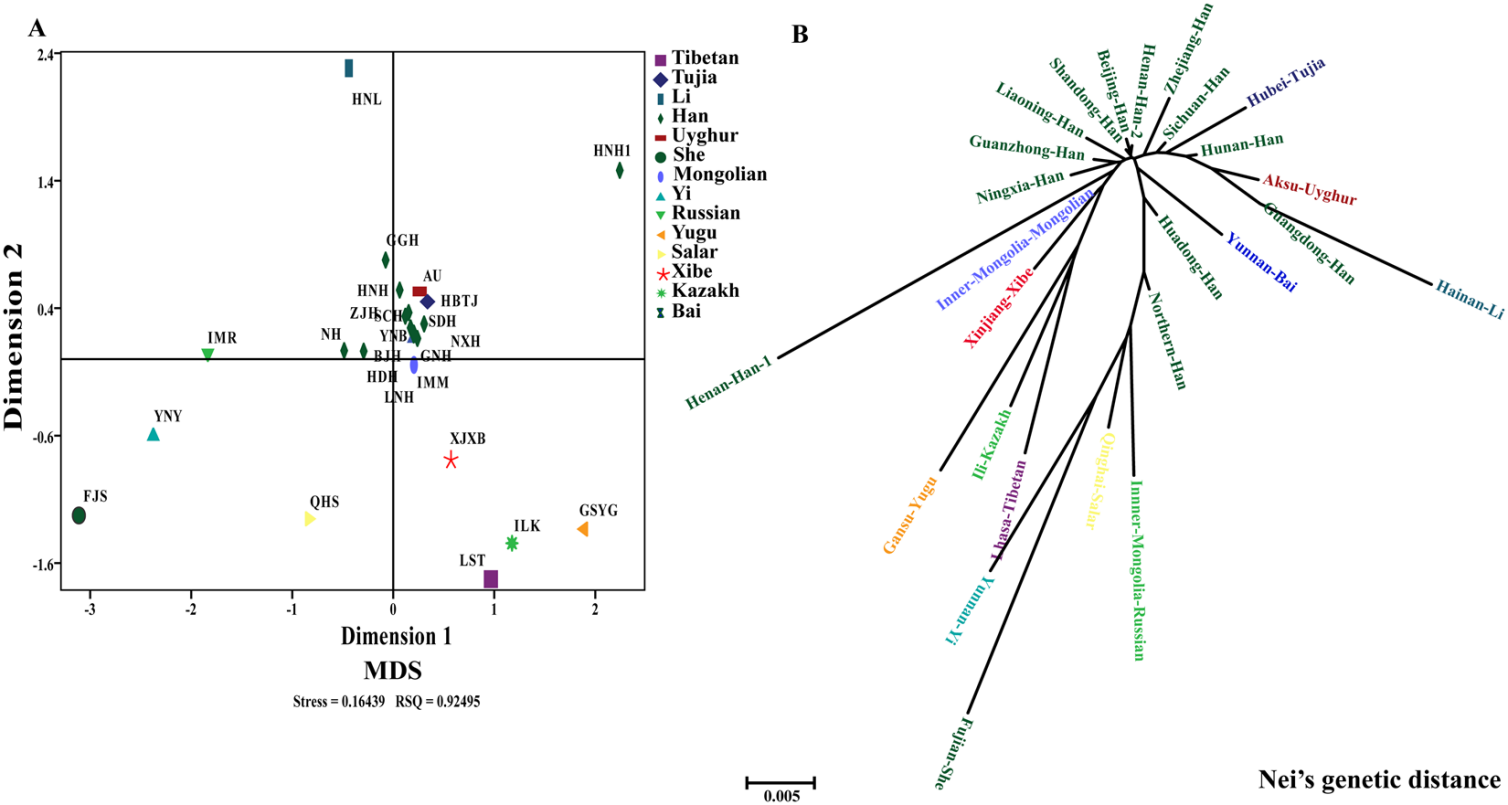
The MDS plot and neighbor-joining phylogenetic tree constructed based on the Nei’s genetic distance matrix.

**Figure 4:**
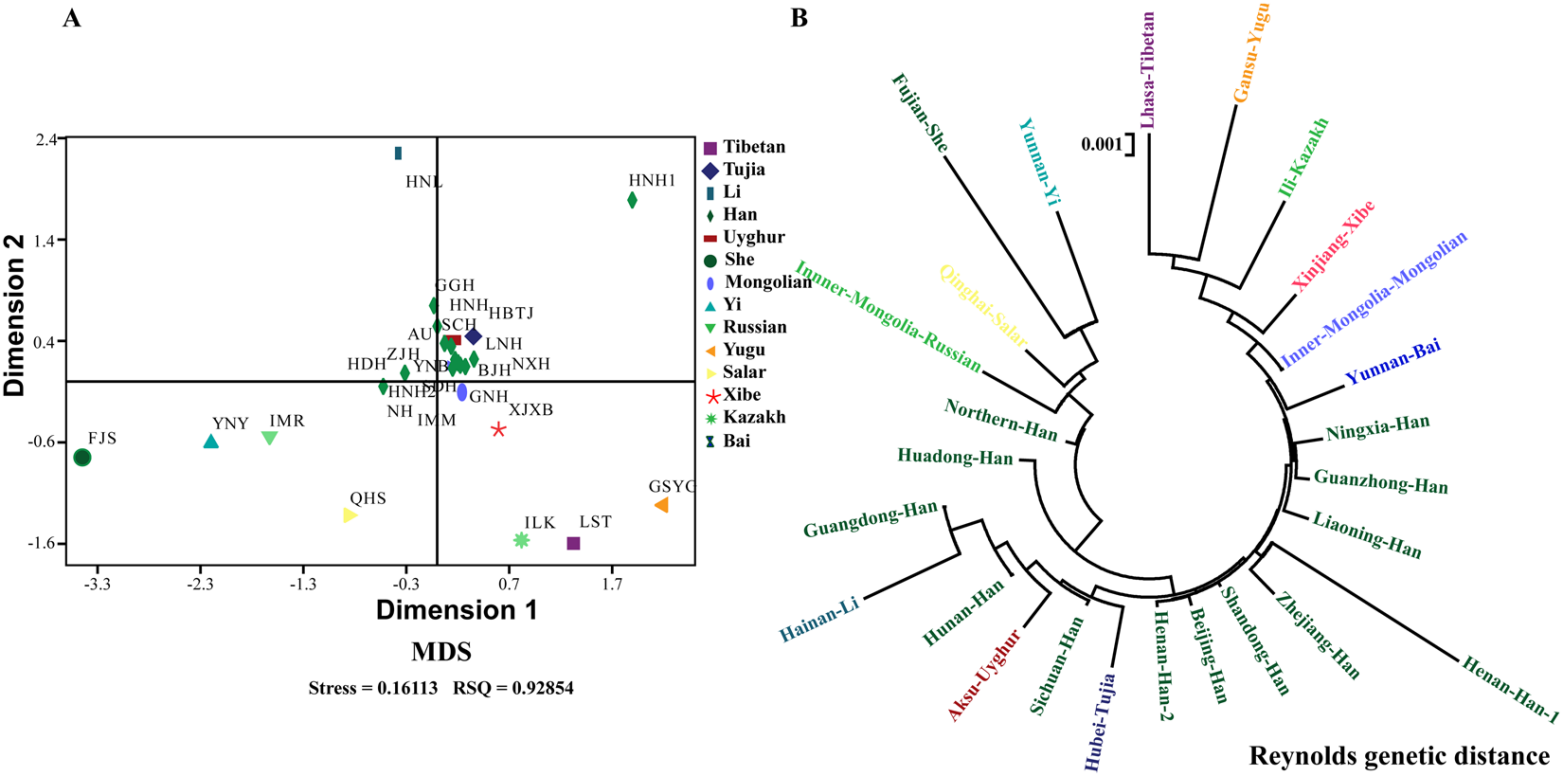
The MDS plot and neighbor-joining phylogenetic tree constructed based on the Reynolds genetic distance.

To validate the population genetic structure pattern, other two genetic distances (Nei’s and Reynolds genetic distances) were employed to reveal the genetic affinity of 26 Chinese populations. The pairwise Nei’s genetic distances are listed in Supplementary Table S7. Fujian She (0.0457), Yunnan Yi (0.0348) and Gansu Yugu (0.0335) were genetically relatively distant from Sichuan Han population, and Hunan Han (0.0036), Henan Han 2 (0.0042) were relatively close to Sichuan Han population. The MDS plot distribution pattern (Fig. 3A) and the phylogenetic structure revealed by the Nei’s genetic distance matrix (Fig. 3B) were in conformity with the characteristics revealed by D_CE_ genetic distance. Additionally, the pairwise Reynolds genetic distances are summarized in Supplementary Table S8. In the MDS plots (Fig. 4A), the isolated populations were consisted with the aforementioned results. In the phylogenetic tree, ethnic minorities were allocated at the both ends of the dendrogram and clustered tightly (Fig. 4B). The Sichuan Han population, falling into the Han groups, was firstly clustered with Aksu Uyghur, and then clustered with Hubei Tujia. We also calculated the Nei’s standard genetic distances among 13 Han Chinese populations and constructed a phylogenetic tree to explore the genetic differentiation. As shown in Supplementary Table S9 and Figure S1, no substructure of the Han population was revealed among 13 Chinese Han populations. Small genetic distances were observed among Han populations (0.0074 ± 0.0103) and slight genetic affinity along geographical position (such as Han population living in Sichuan, Hunan and Guangdong) existed.

In this study, substantial allele frequency distribution difference between Han Chinese populations and ethnic groups was observed, most significantly in She, Yi, Yugu, Tibetan and Kazakh. The results of the MDS and phylogenetic analysis based on three kinds of genetic distances consistently revealed that Yi, She, Salar and Russian had relatively close genetic relationship with each other and may be having a common ancestor. In addition, Tibetan, Kazakh, Yugu, Xibe, Mongolian were clustered in one clade in the three Neighboring-joining trees and had genetic affinity. These aforementioned populations mainly residing in the west of China and showed a geographical affinity and may be descendants from one ancestor. The population genetic stratification was consistent with our previous studies based on X-STRs^47^ and Y-STRs^48,49^. Interestingly, one Uyghur population residing in Aksu kept a genetic affinity with Hunan Han, Hainan Li and Guangdong Han, which may be caused by gene flow in human large-scale migration. In order to further understand population substructure and differentiation within China, more genetic markers, such as ancestry informative SNPs, microhaplotypes and InDels should be used and analyzed in the future genetic researches.

## Conclusions

In this paper, genetic polymorphism data of 368 Han individuals residing in Southwest China were firstly obtained. Results of forensic characteristics indicated that all 21 non-CODIS STRs are highly polymorphic and informative in the Sichuan Han population and can be used as a powerful tool for human identification and forensics. Phylogenetic analysis demonstrated that the Sichuan Han population had a close genetic relationship with the geographically close population (Hunan Han), and significant genetic differences existed between Han populations and ethnic groups (most prominently for Gansu Yugu, Lhasa Tibetan and Fujian She). Furthermore, no significant genetic distinctions between Southern Han and Northern Han but the genetic affinity within North–South gradient changes were observed.

## Methods

### Ethnics standard

Human blood samples were collected upon approval of the Ethics Committee at the Institute of Forensic Medicine, Sichuan University. Informed consent was obtained from all participants. All the experimental procedures and the methods for each procedure were carried out in accordance with the approved guidelines of Institute of Forensic Medicine, Sichuan University. This study was approved by the Ethics Committee of Sichuan University.

### Sample preparation

368 peripheral blood samples were collected from unrelated healthy Han Chinese individuals (132 females and 236 males) recruited from Sichuan Province. All participants were required to have ancestors who had resided in Sichuan Province at least three generations. Human genomic DNA was extracted using PureLink Genomic DNA Mini Kit (Thermo Fisher Scientific) according to the manufacturer’s instructions. The quantity of the DNA template was determined using Quantifiler Human DNA Quantification Kit on a 7500 Real-time PCR System (Thermo Fisher Scientific). Samples were then normalized to 1.0 ng/μL and stored at −20 °C until amplification.

### PCR amplification and genotyping

PCR amplification was performed with 30 PCR cycles following the manufacturer’s protocol on a ProFlex 96-well PCR System (Thermo Fisher Scientific). The PCR system was a 25 μL reaction volume containing 10 μL of Reaction Mix, 5 μL of Primers 21 + 1, 0.75 μL C-Taq and 1 ng of template DNA. The thermal cycling conditions consisted of an initial step at 95 °C for 2 min; followed by 30 cycles of 94 °C for 30 s, 60 °C for 60 s, and 65 °C for 90 s; and a final extension at 60 °C for 60 min.

Amplification products were separated and detected on the Applied Biosystems 3130 Genetic Analyzers using POP-4 polymer and 36 cm capillary array. One microlitre of PCR products or allelic ladder was added to a mixture containing 9.5 μL of deionized Hi-Di formamide and 0.5 of μL AGCU Marker Size-500 (AGCU). The mixture was injected at 1.2 kV for 16 s and electrophoresed at 13 kV for 1550 s with a run temperature at 60 °C. Initial fragment sizing and allele calling were performed using GenoMapper ID v3.20 software with the peak amplitude threshold set at 100 RFUs for all colors.

### Population studies

In order to evaluate the forensic efficiency of this non-CODIS panel for application in Southwest Han, genotype data of 368 unrelated individuals were analyzed. The observed heterozygosity (Ho), expected heterozygosity (He), the exact test of Hardy-Weinberg equilibrium (HWE) and linkage disequilibrium (LD) were estimated and performed using Arlequin 3.5.2.2^50^. Allelic frequencies and forensic parameters, including the polymorphism information content (PIC), power of discrimination (PD), power of exclusion (PE) were calculated using PowerStat V12 spreadsheet (Promega)^51^.

Additionally, genetic similarities and differences between the Sichuan Han population (Southwest China) and other Chinese populations were analyzed. A total of 25 previously investigated Chinese populations containing 9076 individuals were selected^21–45^. The geographic position and detailed information about these 26 Chinese populations are presented in Supplementary Table S1. Analysis of molecular variance (AMOVA) among 26 populations was performed using the Arlequin 3.5.2.2. Three genetic distances (Nei’s genetic distance^52^, Cavalli-Sforza chord distance^53^ and Reynolds genetic distance^54^) between pairwise populations were estimated in the Phylip version 3.695 (http://evolution.genetics.washington.edu/phylip.html) based on allelic frequency distribution. Three genetic distance matrixes were visualized as multidimensional scaling plots in the SPSS software. Principal component analysis (PCA) based on allele frequency distribution was also carried out in the SPSS. Phylogenetic analysis among the aforementioned Chinese populations was conducted in the Mega 6.0^55^ according to the genetic distance matrixes. Three Neighboring-Joining trees were constructed based on different genetic distances.

### Quality control

Control DNA 9947 A (AGCU) and ddH2O were used as positive and negative controls respectively for each batch of genotyping. All experiments were conducted at the Forensic Genetics Laboratory of Institute of Forensic Medicine, Sichuan University, which is an accredited laboratory (ISO 17025), and has been accredited by the China National Accreditation Service for Conformity Assessment (CNAS). We strictly followed the recommendations of Chinese National Standards and Scientific Working Group on DNA Analysis Methods (SWGDAM)^56^.

## Electronic supplementary material

Supplementary Figure S1 and Supplementary Tables S1-S9
