# Supplementary material for: Genetic Diversity and Phylogenetic Differentiation of Southwestern Chinese Han: a comprehensive and comparative analysis on 21 non-CODIS STRs
Source: Sci Rep. 2017 Oct 23;7:13730. doi: 10.1038/s41598-017-13190-w (PMC5653741; doi:10.1038/s41598-017-13190-w)
Supplement: Supplementary file 1 — Supplementary Figure S1 and Supplementary Tables S1-S9 [file 41598_2017_13190_MOESM1_ESM.pdf]

Supplementary Figure S1 and Supplementary Tables S1-S9

Genetic Diversity and Phylogenetic Differentiation of Southwestern Chinese Han: a comprehensive and comparative analysis on 21 non-CODIS STRs

Guanglin He+, Zheng Wang+, Mengge Wang, Yiping Hou*

Institute of Forensic Medicine, West China School of Basic Science and Forensic Medicine, Sichuan University, Chengdu 610041, China

+ These authors contributed equally to this work.

* Corresponding author: Yiping Hou

Institute of Forensic Medicine, West China School of Basic Science and Forensic Medicine, Sichuan University, Chengdu 610041, China

E-mail: profhou@yahoo.com; Phone: +86-28-85501550; Fax: +86-28-85501549.

Supplementary Figure S1. A neighboring-Joining tree among 13 Chinese Han populations.

Northern Hans (Blue color) and Southern Hans (Red color)

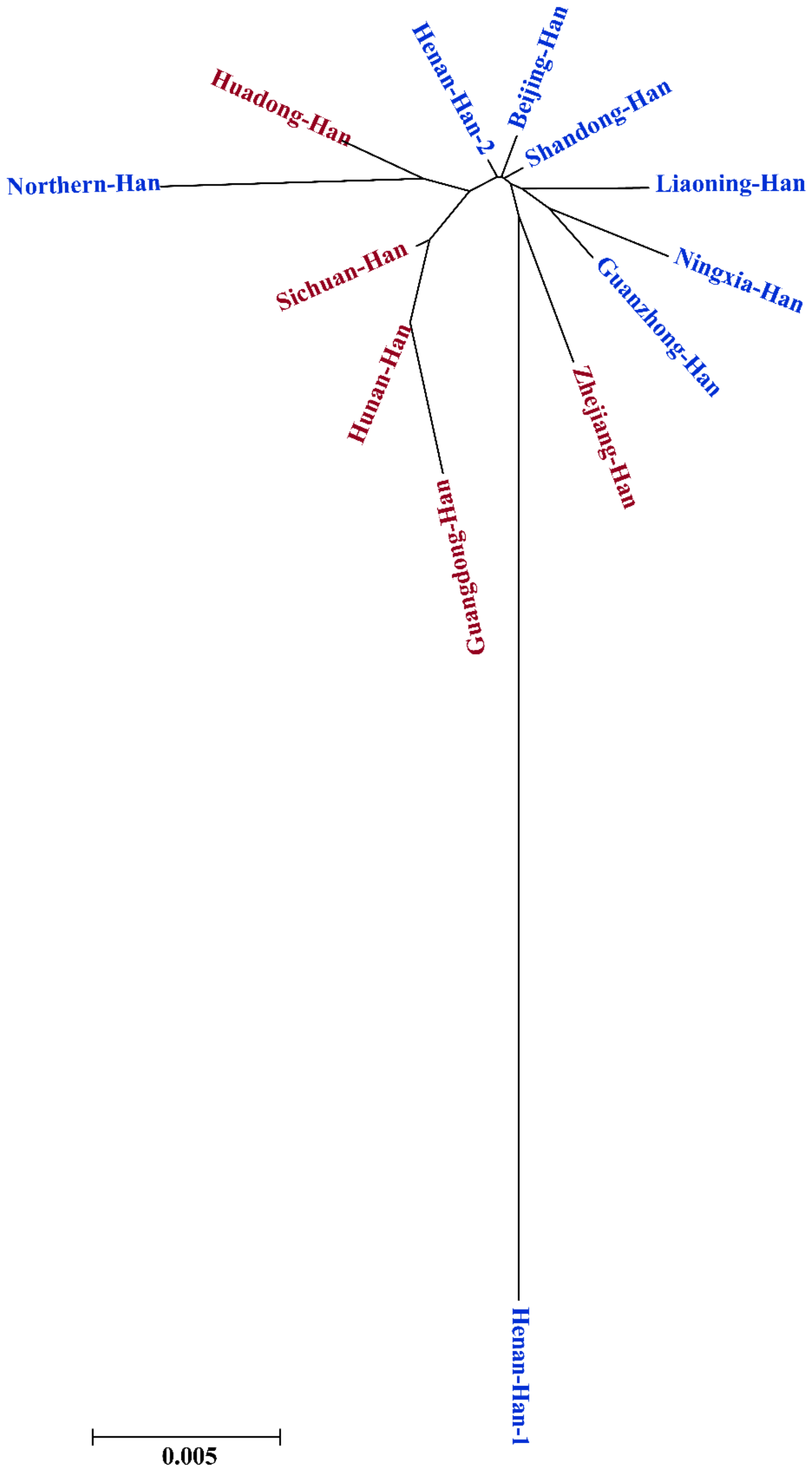

Supplementary Table S1. The detailed information of included studies.

Geographic location	Population	Population name	Abbreviation	No. of subjects
Lhasa, Tibet	Tibetan	Lhasa-Tibetan	LST	104
Qinghai	Salar	Qinghai-Salar	QHS	120
Hubei	Tujia	Hubei-Tujia	HBTJ	107
Yunnan	Bai	Yunnan-Bai	YNB	106
Ningxia	Han	Ningxia-Han	NXH	202
Aksu, Xinjiang	Uyghur	Aksu-Uyghur	AU	502
Ili, Xinjiang	Kazakh	Ili-Kazakh	ILK	114
Fujian	She	Fujian-She	FJS	154
Inner Mongolia	Mongolian	Inner-Mongolia-Mongolian	IMM	523
Northern China	Han	Northern-Han	NH	220
Guanzhong	Han	Guanzhong-Han	GNH	275
Yunnan	Yi	Yunnan-Yi	YNY	110
Hainan	Li	Hainan-Li	HNL	504
Guangdong	Han	Guangdong-Han	GGH	506
Hunan	Han	Hunan-Han	HNH	501
East China	Han	Huadong-Han	HDH	225
Beijing	Han	Beijing-Han	BJH	459
Zhejiang	Han	Zhejiang-Han	ZJH	481
Inner Mongolia	Russian	Inner-Mongolia-Russian	IMR	114
Shandong	Han	Shandong-Han	SDH	1030
Liaoning	Han	Liaoning-Han	LNH	207
Henan	Han	Henan-Han	HNH2	1136
Gansu	Yugu	Gansu-Yugu	GY	180
Xinjiang	Xibe	Xinjiang-Xibe	XJXO	226
Henan	Han	Henan-Han	HNH1	970
Sichuan	Han	Sichuan-Han	SCH	368

Supplementary Table S2. The genotype of 368 Sichuan Han individuals residing in southwest China (132 females and 236 males).

Sample ID	Amelo genin	D10S 1248	D10S 1435	D11S 4463	D12A TA63	D14S 1434	D17S 1301	D18 S853	D19 S433	D1GAT A113	D1S 1627	D1S 1677	D20 S482	D22S 1045	D2S 1776	D2S 441	D3S 4529	D4S 2408	D5S 2500	D6S 1017	D6S 474	D9S 1122
N001	X	11	12	14	16	14	10	11	13	7	13	15	14	15	12	10	14	9	14	17	12	14
	X	13	12	16	19	14	15	13	14	7	13	16	14	17	12	12	15	10	13	16	13	13
N002	X	13	12	15	16	11	12	14	13	7	10	14	14	16	10	11	13	10	17	17	10	14
	X	13	12	15	17	14	13	14	14	13	14	15	14	16	12	12	14	11	11	17	13	12
N003	X	13	13	15	12	10	12	11	13	7	11	14	13	11	9	10	14	8	14	18	8	15
	X	14	14	17	12	14	13	11	13	11	14	15	16	16	12	14	15	10	10	16	13	12
N004	X	12	12	13	12	11	10	11	13	7	13	14	13	15	12	10	13	9	14	20	10	16
	X	16	14	15	12	13	12	11	14.2	7	13	14	13	16	12	14	14	10	10	16	14	10
N005	X	13	10	15	16	14	12	11	13	11	13	15	13	15	12	9.1	14	8	14	23	8	14
	X	15	11	16	17	14	13	14	14.2	12	13	16	14	16	12	14	16	10	10	15	12	11
N006	X	13	11	14	16	12	13	13	13	7	13	15	14	15	9	12	13	10	14	17	8	15
	X	15	13	16	17	14	13	13	15	7	14	16	14	16	10	12	13	10	10	17	13	13
N007	X	14	13	13	12	11	10	11	13	12	13	14	13	15	9	10	13	9	17	17	12	16
	X	15	13	17	12	11	12	13	15.2	12	14	15	14	17	10	12	17	10	12	16	14	11
N008	X	8	12	13	12	13	11	12	13	7	13	13	13	17	10	11	14	8	17	18	12	14
	X	16	14	14	12	15	13	15	13.2	7	13	14	14	17	11	13	14	11	12	15	13	13
N009	X	13	10	13	12	10	12	11	14	7	12	14	15	15	11	12	13	9	18	20	8	16
	X	14	14	14	16	14	12	13	14.2	7	13	15	15	16	13	14	15	10	12	18	13	13
N010	X	13	13	14	12	11	13	11	13.2	7	12	12	14	11	11	11	15	7	14	17	10	14
	X	14	13	15	17	14	15	13	14	11	13	14	14	15	12	11	17	8	11	14	12	12
N011	X	13	11	13	12	13	11	14	12	7	13	14	13	15	11	11.3	13	10	14	21	10	14
	X	14	14	14	17	13	12	15	13	12	14	14	14	16	12	14	15	11	12	17	11	11

N012	X	13	12	14	12	11	12	14	13	7	13	13	13	16	9	10	16	8	14	18	8	14
	X	15	14	15	18	14	12	14	13	11	13	14	14	17	10	11	17	11	10	17	10	10
N013	X	11	13	14	12	13	12	11	13	7	12	14	13	17	9	10	13	9	14	17	8	14
	X	14	15	15	16	14	13	13	13.2	7	13	14	14	17	12	14	15	10	10	15	13	12
N014	X	14	12	13	12	14	11	11	13	7	12	14	14	15	12	10	13	10	14	17	12	14
	X	14	12	15	17	14	12	13	13	12	12	15	14	16	14	11	15	10	13	15	13	13
N015	X	13	13	15	12	11	11	10	13	11	12	13	13	11	9	11	14	9	17	17	8	14
	X	13	13	16	16	15	12	15	16.2	12	13	15	15	15	12	14	15	10	13	15	13	13
N016	X	13	14	15	16	13	10	13	12	7	13	15	14	15	11	11	14	9	18	20	10	15
	X	13	14	16	19	14	13	14	13	11	13	15	16	17	12	11	15	10	12	16	12	11
N017	X	13	13	12	16	11	11	11	14	7	13	14	12	14	11	10	14	8	14	14	11	14
	X	15	15	16	16	14	12	14	14	12	14	15	13	15	12	12	15	11	13	15	13	12
N018	X	13	13	13	16	13	11	11	14	12	13	14	13	17	12	10	14	8	14	18	10	14
	X	15	14	15	16	14	11	14	14.2	12	14	14	14	17	12	11	15	9	11	16	13	13
N019	X	15	12	14	12	11	9	14	13	7	13	15	14	11	11	10	13	10	14	18	10	14
	X	15	14	14	17	13	12	14	13	13	14	15	15	15	11	11	17	10	12	18	13	12
N020	X	13	12	14	16	10	10	13	15	7	13	14	10	17	11	10	13	9	14	17	10	14
	X	13	14	15	16	11	12	14	15.2	12	13	16	14	17	13	11	16	9	12	15	14	11
N021	X	13	12	15	12	13	12	11	14	7	10	14	13	15	9	10	14	8	17	20	10	15
	X	13	14	17	12	14	13	13	16.2	12	10	15	13	17	10	14	17	9	10	15	14	12
N022	X	13	11	13	12	11	13	11	13	11	13	14	10	16	12	11	15	10	17	20	10	14
	X	14	13	14	17	11	13	15	13	12	13	15	13	17	12	14	16	10	13	16	13	13
N023	X	13	11	13	16	13	12	11	13.2	7	12	15	13	15	11	10	15	8	14	18	8	12
	X	15	13	13	17	14	12	13	15.2	13	13	15	14	16	12	11	16	9	10	17	14	12
N024	X	15	11	13	12	11	12	11	13	7	13	13	14	16	11	11	14	10	17	20	10	15
	X	15	14	15	17	14	12	14	13	7	14	15	15	16	12	11	16	12	12	17	12	12

N025	X	12	11	14	12	10	11	12	13	7	12	12	13	11	9	10	14	9	14	17	8	15
	X	13	14	15	18	13	12	15	15.2	11	12	15	14	15	12	11	16	9	10	16	11	11
N026	X	14	12	14	17	13	12	11	13	7	13	14	12	15	9	11.3	14	9	14	18	8	14
	X	15	12	15	18	13	13	11	15.2	11	13	15	14	17	14	12	15	12	8	15	14	13
N027	X	13	12	14	17	13	11	11	13	7	13	14	13	11	11	11	15	9	17	17	12	14
	X	15	13	15	17	14	12	11	15	7	13	14	15	15	12	11	15	9	12	17	13	13
N028	X	13	11	15	12	14	13	11	13	7	13	14	14	15	11	10	14	9	18	18	10	15
	X	13	13	15	16	14	13	13	15.2	11	13	15	14	17	12	14	17	9	12	17	12	11
N029	X	13	9	16	17	14	11	11	14	12	12	14	14	11	10	11	14	9	18	20	12	15
	X	15	13	16	18	14	12	15	14.2	12	14	15	15	16	12	14	15	9	13	17	13	12
N030	X	13	11	14	17	13	11	11	14	7	13	12	13	16	12	10	13	9	14	14	8	14
	X	14	13	16	18	14	13	14	15.2	7	14	14	13	17	13	11	14	10	10	14	13	10
N031	X	13	13	15	12	13	11	11	13	7	12	13	14	15	10	10	14	10	14	18	8	15
	X	15	14	16	17	14	13	11	16.2	11	13	14	14	17	11	12	16	10	10	16	13	12
N032	X	13	10	14	14	10	11	11	15	12	13	14	15	11	11	10	14	11	17	18	11	15
	X	13	14	15	18	14	12	15	15	12	15	15	15	17	11	12	14	11	12	15	12	12
N033	X	13	11	14	12	13	9	11	14	7	14	12	13	17	11	10	13	10	18	18	10	14
	X	14	12	16	16	14	13	15	15.2	7	14	14	14	17	13	11	13	11	10	16	13	11
N034	X	14	11	14	17	14	12	12	13	7	14	15	10	11	9	10	13	9	14	18	12	14
	X	16	12	14	18	14	13	14	14	7	14	15	13	16	11	12	15	10	12	15	12	11
N035	X	14	12	14	12	13	10	11	13	7	14	13	13	11	9	12	13	9	14	18	8	14
	X	15	14	14	12	14	14	11	15.2	12	15	13	15	16	13	12	13	10	10	15	13	12
N036	X	12	12	14	12	14	11	11	14	11	12	13	12	15	12	10	13	9	17	18	10	15
	X	13	12	14	17	14	13	13	15.2	11	13	14	14	17	12	11	14	11	12	16	12	11
N037	X	13	11	13	17	10	12	11	13.2	7	13	14	13	11	11	12	13	10	14	18	10	14
	X	15	11	14	18	13	12	14	15	13	14	16	14	15	11	14	14	10	12	15	13	12

N038	X	13	12	14	16	13	11	11	14	7	13	14	12	11	11	10	14	9	14	18	10	16
	X	15	13	14	17	14	12	14	14	12	13	15	13	11	11	11	15	10	13	16	12	11
N039	X	13	11	15	12	11	12	11	13	7	13	14	14	11	11	10	15	9	14	14	8	14
	X	14	14	15	12	14	12	12	14	11	13	14	15	15	11	12	16	9	13	17	13	11
N040	X	14	11	14	12	14	12	13	14	11	10	14	12	11	10	14	13	11	14	17	10	14
	X	16	13	14	18	14	13	13	14.2	12	13	15	14	17	11	14	14	12	10	18	13	12
N041	X	13	12	14	12	10	12	14	13	7	13	14	12	15	9	10	16	9	14	18	12	17
	X	14	13	14	16	13	12	14	16	10	13	16	13	17	13	11	16	10	12	17	13	11
N042	X	14	10	12	12	13	11	11	14	12	13	14	13	11	9	11	13	9	14	20	10	15
	X	14	13	14	17	14	12	13	15	12	13	16	13	16	11	12	16	10	12	17	12	12
N043	X	13	12	14	14	13	12	13	14	7	12	14	16	11	9	10	15	9	14	18	8	14
	X	13	12	15	18	14	12	14	15.2	13	13	15	16	16	11	10	17	10	8	15	13	11
N044	X	14	12	9	16	13	11	11	13	7	13	15	13	15	12	12	15	8	14	18	10	14
	X	15	12	13	16	14	11	15	14	12	13	15	15	16	14	12	15	9	10	16	13	12
N045	X	14	11	13	12	11	11	11	13	7	13	14	12	14	11	12	15	9	14	18	8	14
	X	15	13	15	18	14	12	14	14	7	13	15	13	15	12	14	15	11	12	15	13	12
N046	X	13	11	11	12	14	13	11	13	12	13	14	13	11	10	12	13	8	17	18	8	15
	X	15	12	13	18	14	13	13	14	12	13	15	14	17	12	14	16	9	12	16	13	10
N047	X	15	13	13	16	11	10	11	13	7	13	14	12	15	13	12	13	9	14	14	12	17
	X	16	14	16	16	11	12	13	13	7	14	16	14	16	13	12	17	10	14	18	13	11
N048	X	13	11	12	12	14	12	11	13	7	13	14	13	17	12	10	14	10	14	14	12	15
	X	15	12	14	17	14	13	14	15	7	15	15	13	17	13	11	15	10	12	17	13	12
N049	X	12	12	12	14	11	12	11	13	7	14	14	13	15	12	11	14	8	14	18	12	14
	X	13	12	14	17	14	13	14	13	12	15	15	15	17	12	14	15	10	13	15	13	12
N050	X	13	13	12	17	11	10	11	13	12	12	14	12	11	11	14	13	8	14	17	10	14
	X	13	14	14	18	13	14	13	14	12	14	14	12	17	14	15	15	11	10	14	13	11

N051	X	13	12	12	17	11	10	11	14	7	13	13	12	11	11	10	13	8	14	14	10	14
	X	14	13	15	18	13	13	13	14.2	12	14	14	13	17	11	15	14	11	12	14	13	11
N052	X	13	12	13	17	13	11	13	13	11	13	15	13	15	10	11	14	9	17	20	8	15
	X	14	12	15	18	14	12	14	13	12	13	15	14	15	12	12	14	11	10	15	13	11
N053	X	14	12	16	16	13	9	14	13	11	13	14	13	15	9	11	13	10	17	18	12	15
	X	14	12	16	17	14	13	14	15.2	11	14	14	13	16	12	11.3	15	10	12	16	13	13
N054	X	14	12	13	12	14	11	11	14	12	12	15	13	16	11	11	14	9	18	18	12	14
	X	14	13	14	17	14	12	11	15.2	12	13	15	14	16	11	14	16	10	13	14	11	11
N055	X	12	12	14	12	13	9	11	12	7	13	14	13	15	11	10	13	8	14	18	8	14
	X	17	13	15	12	14	13	11	16.2	13	13	14	16	16	11	11	14	11	10	18	13	12
N056	X	14	14	14	14	11	12	13	13	7	13	14	13	16	12	10	14	9	14	17	10	14
	X	14	14	15	17	13	13	14	14.2	7	13	14	14	17	12	12	14	10	12	17	13	13
N057	X	13	12	13	17	11	12	13	14	7	13	14	13	15	9	12	14	8	14	14	10	16
	X	15	12	14	18	13	13	13	15.2	7	13	14	14	16	12	12	14	10	12	17	12	11
N058	X	12	12	14	17	14	11	14	14	7	13	14	10	15	11	11	13	9	14	18	10	15
	X	15	12	14	17	14	13	15	15.2	12	13	14	14	16	12	12	15	11	14	17	13	10
N059	X	13	12	13	17	14	12	11	13.2	7	12	15	15	15	11	12	13	8	17	17	10	14
	X	14	15	14	17	14	13	15	14.2	11	14	15	16	16	12	12	15	10	12	14	13	13
N060	X	13	12	12	17	13	11	11	14	7	13	15	14	11	10	10	14	8	14	18	11	14
	X	16	13	17	17	14	15	11	15	12	14	15	15	17	12	12	14	10	13	15	12	11
N061	X	13	13	15	12	11	12	11	14.2	7	12	13	13	16	10	11	13	10	14	18	10	15
	X	13	14	16	18	14	12	13	14.2	12	13	14	15	17	11	13	13	10	12	15	13	12
N062	X	13	13	13	12	10	11	11	12	7	14	13	11	15	9	10	13	9	17	20	8	15
	X	14	13	13	17	13	13	12	15.2	12	14	15	13	16	10.1	10	13	10	10	16	15	12
N063	X	13	13	13	17	11	11	11	13	7	13	13	14	16	9	11	14	8	14	18	10	16
	X	15	13	14	17	14	12	14	15	13	14	16	14	16	12	15	15	10	10	17	13	11

N064	X	14	11	13	12	11	12	11	13	7	10	14	13	16	12	11.3	13	11	17	17	10	14
	X	16	13	14	17	14	13	14	14	7	12	15	15	16	12	12	16	12	12	15	13	12
N065	X	12	8	13	17	11	12	13	13	12	10	15	13	11	11	11	13	8	14	17	8	14
	X	14	12	16	18	12	12	14	15.2	12	13	15	13	15	14	11	13	8	10	15	13	13
N066	X	13	11	13	17	11	11	11	13	12	12	14	12	15	11	10	14	10	17	23	8	14
	X	14	14	14	17	14	13	13	15.2	12	13	14	13	16	11	11	15	10	12	16	16	13
N067	X	13	12	15	16	13	11	11	14	7	13	14	13	11	10	10	14	9	17	17	10	14
	X	13	14	15	16	13	12	14	16.2	7	13	15	13	16	11	14	15	10	12	15	11	11
N068	X	14	8	14	17	14	10	13	13	7	13	14	15	16	11	11	13	8	18	20	10	14
	X	15	12	16	17	14	11	13	14	12	14	15	16	17	12	14	17	9	10	15	12	10
N069	X	13	8	14	12	14	11	13	14	7	13	15	15	11	11	11	13	8	14	18	10	14
	X	15	13	16	17	14	12	14	14	12	14	15	15	17	12	11	15	9	10	15	13	12
N070	X	12	12	14	12	14	12	14	13	7	12	15	14	15	12	11	13	8	14	18	12	15
	X	14	13	14	16	14	12	15	14	12	13	16	14	17	12	12	17	8	12	15	13	12
N071	X	13	11	13	16	10	10	14	14	7	10	13	14	11	9	10	15	8	14	18	12	14
	X	13	12	16	17	13	12	14	14	11	14	15	16	16	12	11	15	10	13	15	11	10
N072	X	15	12	13	16	10	11	11	13	7	12	14	13	11	11	12	14	8	14	17	10	15
	X	16	14	15	17	13	14	13	14	13	14	15	15	16	12	12	15	9	12	17	12	12
N073	X	13	8	14	16	13	13	11	13	7	13	15	11	15	10	11	15	9	14	17	10	14
	X	13	12	16	17	14	14	11	14	11	14	16	14	17	12	13	15	10	12	15	12	12
N074	X	13	12	13	16	14	12	11	14	7	14	15	14	15	11	10	13	9	14	17	8	14
	X	14	13	14	17	14	14	15	16.2	7	14	15	14	16	12	12	13	11	8	15	14	13
N075	X	13	12	14	12	13	9	11	13	7	13	14	13	11	9	12	14	9	17	18	8	15
	X	15	13	15	12	14	10	11	13	12	14	15	15	15	10	12	14	9	10	16	13	13
N076	X	14	10	13	12	14	13	11	13	12	14	14	14	16	11	10	15	9	14	14	8	14
	X	14	12	16	12	14	14	13	15.2	12	14	14	16	16	11	14	16	10	12	17	13	13

N077	X	13	11	15	12	12	11	11	14.2	7	13	14	13	15	12	10	15	8	14	17	8	15
	X	15	12	16	16	14	12	13	16.2	12	13	15	14	16	12	10	16	10	10	16	13	10
N078	X	13	13	14	12	11	11	11	13	11	12	14	13	11	9	11	15	8	14	18	10	14
	X	15	13	15	12	14	12	14	13	11	13	15	14	15	11	11	16	9	14	15	13	12
N079	X	15	11	13	12	10	12	13	14	7	12	14	14	16	10	12	13	8	18	18	8	14
	X	15	14	14	18	13	13	13	14	11	14	15	14	16	10	12	14	10	12	16	13	13
N080	X	13	11	15	12	14	12	11	12	7	13	13	14	11	9	10	14	10	17	19	10	14
	X	15	14	16	17	14	13	15	14.2	11	14	13	14	17	11	11	15	12	12	16	12	12
N081	X	13	8	13	17	10	9	14	13	7	12	14	12	15	12	10	13	9	14	14	8	15
	X	13	12	16	18	13	12	14	15	12	14	14	15	15	12	10	16	9	13	16	13	10
N082	X	13	13	13	17	10	13	11	12	7	12	14	13	11	10	11	13	9	14	14	10	14
	X	14	14	14	17	14	14	13	13	12	14	15	14	16	11	14	16	11	10	15	12	12
N083	X	13	12	13	12	13	12	13	13	7	13	14	13	15	11	11	13	9	18	18	12	14
	X	15	13	15	16	14	12	14	14	13	13	15	14	16	12	12	15	10	12	15	12	12
N084	X	13	12	14	12	13	11	11	13	11	13	14	13	16	9	12	15	9	17	18	8	14
	X	13	14	16	12	14	13	11	14	12	13	15	14	17	12	12	15	10	8	15	12	12
N085	X	12	11	14	16	13	11	13	13	7	12	15	13	15	9	10	13	8	17	20	10	15
	X	13	14	16	17	15	12	13	15.2	12	14	16	14	17	11	14	14	10	12	16	11	10
N086	X	13	13	15	17	13	10	11	13	7	13	15	13	15	10	10	13	9	14	17	10	14
	X	15	14	15	18	14	14	14	13.2	12	13	16	15	17	11	14	15	9	10	16	13	12
N087	X	13	12	15	12	11	11	11	13	7	13	14	16	16	9	11	13	10	14	20	12	15
	X	13	14	15	18	14	12	15	15	13	13	15	16	17	12	14	16	10	12	18	13	10
N088	X	13	12	14	17	10	12	13	14	7	13	14	13	11	11	10	13	10	14	14	10	15
	X	14	13	14	18	11	12	13	15.2	7	13	14	16	15	13	11	15	11	13	15	13	12
N089	X	13	10	15	12	13	12	11	14.2	7	13	13	13	11	9	10	13	10	14	17	8	15
	X	16	12	16	12	14	13	13	15.2	12	14	14	13	16	13	10	16	12	10	16	13	13

N090	X	12	12	13	12	13	12	11	13	7	13	14	15	11	12	10	15	8	14	17	10	14
	X	14	12	13	14	13	13	15	15.2	11	14	15	15	15	12	11.3	15	10	12	14	15	12
N091	X	14	10	15	17	13	12	11	12.2	7	13	15	14	15	12	9	13	10	14	14	10	14
	X	15	12	16	18	14	12	12	13	12	13	15	15	16	12	9	14	10	13	14	13	12
N092	X	12	12	13	12	13	12	11	14.2	7	13	14	13	16	10	10	16	9	17	20	10	14
	X	15	13	14	17	13	12	15	15.2	12	14	14	13	16	11	12	16	11	12	15	13	12
N093	X	13	12	14	14	14	12	11	12	7	11	14	13	15	9	10	15	9	14	18	8	14
	X	15	14	15	18	15	12	14	14.2	12	14	15	14	16	11	11	19	11	10	15	12	12
N094	X	14	13	14	16	13	11	11	14	12	13	14	10	11	12	10	13	10	14	18	10	14
	X	14	14	14	16	14	12	11	15.2	12	13	15	14	16	13	10	13	11	10	15	13	13
N095	X	13	8	13	16	11	11	13	14.2	7	13	14	13	11	11	11.3	14	10	17	17	8	14
	X	16	13	14	17	14	13	13	14.2	12	14	14	14	15	11	12	15	12	10	15	12	12
N096	X	15	13	12	12	10	12	10	13	7	14	13	14	15	11	12	14	9	14	17	12	15
	X	16	14	15	17	14	12	13	14	11	14	14	15	17	11	14	16	9	12	16	13	13
N097	X	13	11	15	12	13	13	12	15.2	7	13	13	13	15	9	11	14	8	17	17	8	14
	X	14	12	16	12	14	13	13	15.2	12	13	15	14	17	12	11	16	10	10	15	13	11
N098	X	14	11	13	17	12	11	11	13	12	14	13	13	15	12	11	13	9	14	17	8	15
	X	17	11	14	17	14	14	15	13	12	14	14	13	15	14	13	16	9	10	16	12	12
N099	X	13	13	13	17	13	11	11	14	12	12	14	12	16	11	10	14	10	18	18	10	15
	X	15	14	16	18	14	12	13	15	12	13	15	13	17	12	10	15	11	13	17	14	13
N100	X	11	12	13	12	13	12	10	14.2	7	13	15	12	11	11	10	15	9	14	17	10	15
	X	16	15	15	12	14	13	11	15.2	11	14	15	14	11	12	12	15	9	12	16	12	12
N101	X	13	13	14	12	14	12	11	13	7	12	14	10	15	11	11	13	9	17	18	8	14
	X	15	13	15	16	14	12	14	13	7	13	15	15	17	11	11.3	15	10	11	15	14	11
N102	X	13	13	13	12	13	11	11	12	12	13	15	14	11	9	12	13	9	14	14	12	14
	X	13	13	13	16	14	11	14	13	12	13	15	14	15	14	12	14	10	12	14	14	12

N103	X	13	13	13	16	13	11	10	12	12	12	13	14	15	11	12	13	9	14	14	8	15
	X	14	13	15	16	14	12	11	13	12	13	14	14	17	11	12	14	10	12	17	13	12
N104	X	14	12	14	12	13	12	11	12	7	13	13	13	11	11	10	15	8	14	14	8	14
	X	15	14	14	17	14	12	13	14.2	7	13	14	14	15	11	12	16	12	8	17	13	13
N105	X	12	12	16	17	13	12	14	13	7	12	14	13	11	11	12	13	10	14	18	10	14
	X	15	13	16	17	14	12	14	14	12	13	16	14	11	13	12	15	10	12	15	13	13
N106	X	13	13	13	12	13	9	11	12	7	12	14	12	11	11	10	15	9	14	14	8	14
	X	14	14	13	17	13	12	11	15.2	12	13	14	13	15	12	10	17	10	8	14	13	13
N107	X	13	12	12	16	11	11	11	13	7	13	14	14	11	9	11	13	8	14	18	10	14
	X	16	12	13	16	11	12	13	14	12	13	15	15	15	11	11	13	8	10	15	13	12
N108	X	12	13	14	14	10	11	11	13	7	12	14	14	15	10	12	15	9	17	20	11	14
	X	14	14	15	18	14	13	12	16	12	13	15	15	19	12	14	16	10	12	17	12	12
N109	X	13	11	13	12	13	13	11	14	7	13	14	13	11	12	10	15	9	14	18	8	14
	X	15	12	14	12	13	13	14	15	7	13	14	14	15	12	15	15	9	10	15	14	11
N110	X	12	13	15	16	13	9	11	13	11	13	14	13	11	11	10	13	8	14	17	10	15
	X	13	13	15	17	14	11	13	13.2	12	13	14	14	11	12	11.3	15	10	12	15	13	12
N111	X	12	12	16	12	13	11	11	14	12	12	13	13	16	11	11	15	8	18	18	10	14
	X	16	13	16	17	13	12	13	15.2	12	15	15	13	16	13	11	16	9	13	15	13	12
N112	X	13	11	14	12	13	13	11	12	7	13	14	10	11	11	10	14	8	14	18	10	15
	X	13	12	16	14	14	13	11	14	12	14	15	14	15	12	10	16	9	12	15	13	12
N113	X	13	12	16	16	13	11	11	13.2	12	12	14	13	15	11	10	15	8	14	18	8	17
	X	13	14	16	17	14	12	13	14	12	12	14	15	17	12	14	15	10	13	17	13	12
N114	X	13	12	14	12	13	12	11	13	7	13	14	12	14	9	11	14	9	14	17	10	14
	X	13	13	14	12	14	12	15	15	12	14	15	14	17	11	12	16	10	10	16	12	12
N115	X	14	12	13	16	13	13	14	14	7	14	14	13	15	11	10	15	9	14	14	8	14
	X	15	13	15	17	14	13	16	15.2	7	14	15	13	17	12	11	16	9	13	15	13	12

N116	X	13	11	15	12	13	13	12	15.2	7	10	13	13	15	9	11	14	8	17	17	8	14
	X	14	12	16	12	14	13	13	15.2	12	13	15	14	17	12	11	16	10	10	15	13	11
N117	X	13	12	14	16	11	12	11	14	7	13	14	11	16	11	10	14	8	14	14	12	14
	X	13	12	15	16	13	12	14	14.2	7	13	15	14	16	12	12	15	10	12	17	13	13
N118	X	15	13	12	12	10	12	13	13	11	13	14	13	15	11	12	14	9	17	20	12	14
	X	15	13	13	16	13	12	14	13	12	14	16	14	17	12	12	14	10	12	15	13	9
N119	X	13	8	13	16	10	12	11	14	7	13	14	12	15	11	10	15	8	14	17	8	15
	X	13	14	15	18	13	12	14	15	12	14	14	15	15	12	10	16	9	10	15	13	12
N120	X	13	12	13	12	13	11	13	13	7	10	14	13	15	10	10	15	8	17	20	10	15
	X	13	14	15	12	14	13	13	15.2	7	10	15	13	17	11	11.3	17	10	11	15	12	12
N121	X	15	12	14	17	11	12	13	14	7	13	15	14	15	11	10	13	10	14	20	10	15
	X	16	16	17	18	13	15	13	15	7	14	15	16	16	12	11	15	10	10	17	13	12
N122	X	12	12	13	12	13	10	11	12	7	13	14	14	16	9	10	14	8	14	18	8	15
	X	16	14	16	16	13	12	12	13	7	13	14	15	16	10	11	15	9	8	17	12	12
N123	X	14	11	13	12	11	11	11	15	11	12	14	14	16	10	11	14	9	14	18	8	15
	X	17	14	15	16	14	13	11	15.2	12	13	15	15	16	13	11	15	12	10	15	11	11
N124	X	14	12	12	16	13	10	13	14	7	13	14	14	15	11	10	15	9	17	18	12	14
	X	14	12	14	17	14	12	13	14.2	7	13	14	15	17	12	11	16	9	12	15	13	11
N125	X	15	10	12	12	13	12	14	14	7	14	14	13	15	10	11	13	8	14	18	8	14
	X	16	12	16	17	14	12	14	14.2	7	14	14	14	15	11	14	14	8	10	15	12	12
N126	X	13	13	14	12	14	12	11	13	7	12	14	13	17	9	10	16	10	17	18	10	14
	X	14	13	15	17	16	12	14	15.2	7	13	14	15	18	12	11	16	11	12	16	12	11
N127	X	13	10	13	17	14	11	11	13	7	13	14	13	15	9	11	14	8	14	20	10	14
	X	13	12	14	17	14	13	12	15	12	14	15	14	16	10	12	14	10	10	16	12	12
N128	X	14	11	13	12	13	12	13	13	7	13	11	13	15	9	11	14	9	14	14	12	14
	X	14	14	16	17	14	14	14	14	12	14	17	14	17	11	11.3	16	10	13	17	14	13

N129	X	13	13	12	16	11	10	11	12.2	7	12	13	14	15	11	10	14	10	17	17	12	15
	X	16	15	16	17	14	13	11	14	7	13	16	15	15	12	11	15	11	13	15	13	11
N130	X	13	12	13	17	13	12	11	13	7	13	12	13	15	11	10	15	8	14	17	10	15
	X	14	13	14	18	14	13	13	13	11	14	14	14	16	12	14	16	10	13	17	12	10
N131	X	13	12	12	12	10	12	11	15	7	13	13	13	11	11	11	15	9	14	18	13	14
	X	15	12	15	12	14	13	15	15.2	12	14	15	14	16	15	12	15	9	13	15	13	13
N132	X	14	11	15	16	10	12	11	13.2	11	14	10	10	11	12	11	13	10	14	18	10	14
	X	16	11	15	17	14	12	12	15	12	14	15	14	11	12	13	14	12	12	14	13	11
N133	X	13	12	15	16	10	12	11	12	11	10	13	14	11	11	11	14	11	17	17	8	14
	Y	14	13	16	17	13	13	14	13	12	13	14	15	15	11	12	15	11	14	16	13	12
N134	X	13	12	13	12	13	10	14	14	7	14	14	14	15	12	10	15	10	17	18	10	14
	Y	13	15	14	17	14	13	14	16.2	11	14	14	15	16	12	12	15	10	12	15	14	11
N135	X	14	12	13	12	13	13	11	14.2	7	13	14	10	11	11	10	13	11	18	20	10	14
	Y	16	14	15	12	13	14	14	15.2	7	14	14	15	15	13	11	15	12	13	15	13	13
N136	X	13	12	15	12	14	11	11	15	12	13	15	15	11	9	10	14	8	14	18	8	15
	Y	14	13	17	17	14	13	11	16.2	12	14	15	16	17	11	12	16	10	10	15	12	10
N137	X	12	12	13	12	11	9	11	14.2	11	13	14	14	16	9	10	14	8	14	17	8	15
	Y	15	12	15	12	14	12	11	15.2	12	14	15	15	17	12	11	15	9	10	16	13	10
N138	X	15	12	14	12	10	12	11	14	12	13	14	14	11	11	10	15	9	14	18	8	16
	Y	15	12	15	12	13	13	13	16	12	13	15	15	15	12	13	16	9	14	17	14	11
N139	X	13	12	12	12	10	12	13	13	7	13	15	14	11	11	10	15	9	14	18	10	14
	Y	13	12	14	16	11	13	13	13.2	13	13	15	16	11	12	11	16	11	13	15	12	11
N140	X	13	11	14	12	13	10	10	14.2	7	13	13	13	16	9	10	13	10	17	18	10	15
	Y	15	13	15	12	13	12	11	16.2	7	13	14	14	17	12	11	13	11	10	15	13	12
N141	X	12	11	13	12	11	11	13	14	7	12	15	13	16	10	11.3	15	9	17	20	10	15
	Y	16	13	13	12	15	12	13	15.2	11	13	17	13	17	12	12	17	10	12	15	12	12

N142	X	14	13	13	12	13	11	13	13	7	13	14	14	11	12	11	13	9	14	18	8	14
	Y	15	14	16	17	14	11	13	14.2	11	14	14	15	16	13	14	16	10	10	14	14	12
N143	X	13	12	16	12	11	12	13	13	11	13	13	14	16	11	12	14	8	17	17	8	15
	Y	14	13	16	17	13	13	14	14	12	14	15	14	17	12	14	14	9	10	16	13	13
N144	X	13	12	14	12	10	13	11	13	7	14	14	13	16	12	11	15	10	14	17	8	14
	Y	13	14	15	12	13	13	14	13	7	14	14	13	16	13	12	16	11	10	14	13	13
N145	X	14	11	13	12	10	11	14	14	12	13	14	13	15	11	11	14	9	14	14	8	15
	Y	14	14	13	12	13	13	14	15.2	12	13	14	16	17	13	11	14	9	10	15	14	13
N146	X	14	12	14	16	13	12	13	13	7	13	14	12	17	11	10	13	8	14	18	8	14
	Y	14	13	14	17	14	12	14	14	11	14	14	14	17	11	12	15	9	13	17	13	12
N147	X	12	13	13	18	10	12	11	13	7	13	14	13	11	11	10	15	8	14	18	10	15
	Y	15	14	14	18	14	13	11	14	12	14	15	14	18	12	11	17	8	10	16	13	11
N148	X	13	12	12	12	10	12	13	12	7	13	15	14	15	12	12	14	9	14	20	10	15
	Y	16	14	13	15	14	12	14	14.2	7	13	15	15	15	13	14	15	10	12	16	13	12
N149	X	12	11	15	17	14	12	11	13.2	7	13	15	14	15	13	11	14	9	14	14	10	14
	Y	15	12	15	17	14	14	11	14.2	12	13	15	15	16	14	11	14	10	12	17	12	12
N150	X	13	11	14	17	11	11	11	13	7	13	14	13	11	12	10	13	8	14	14	12	16
	Y	14	13	15	17	13	12	14	13	11	14	16	14	17	13	11	15	9	12	16	13	13
N151	X	14	12	13	17	13	12	11	14	7	13	14	13	11	9	10	14	9	14	17	8	15
	Y	14	13	15	17	14	12	11	16	7	15	16	14	11	11	14	16	9	12	16	12	11
N152	X	12	11	14	17	10	12	13	13	11	14	14	14	11	9	10	14	8	17	18	10	14
	Y	14	12	15	17	14	13	14	13	12	14	15	15	17	11	11	15	10	12	14	12	11
N153	X	13	11	13	14	14	12	13	14	7	13	13	15	15	9	11.3	13	8	17	18	8	15
	Y	15	13	15	17	15	12	13	14	12	14	13	15	17	13	12	14	11	10	17	12	10
N154	X	13	12	15	17	10	11	13	14	11	14	14	13	16	10	10	13	8	14	18	7	14
	Y	16	13	15	17	14	12	13	14	12	14	16	15	17	11	14	14	10	8	14	13	12

N155	X	12	12	13	17	11	12	11	13	7	13	14	13	15	11	10	16	9	17	20	8	16
	Y	13	14	16	18	11	12	15	15.2	7	14	15	13	16	12	11.3	16	9	10	17	13	12
N156	X	14	12	15	12	13	13	13	13	11	14	14	14	16	11	11	15	10	14	17	8	15
	Y	15	13	15	17	14	13	13	13	12	14	14	15	16	11	11	16	10	10	17	13	12
N157	X	12	11	13	12	13	11	11	14	7	14	14	14	15	12	12	13	10	14	17	8	15
	Y	13	14	15	17	14	13	12	15.2	12	14	15	16	18	12	14	15	10	8	15	13	12
N158	X	13	12	16	17	10	12	11	13	7	14	14	12	11	11	11.3	13	8	18	20	12	16
	Y	14	13	16	18	14	13	14	13	11	14	15	14	16	12	14	14	8	13	17	11	11
N159	X	14	12	14	12	14	12	13	15	7	12	14	13	11	12	11	14	8	14	17	8	14
	Y	15	13	15	17	14	13	14	15.2	7	14	14	14	15	13	11	15	9	10	15	13	11
N160	X	13	13	14	12	13	12	12	13	7	13	13	13	16	11	10	14	9	14	17	10	15
	Y	13	14	15	18	13	13	13	15.2	12	13	15	14	17	13	12	14	10	10	15	14	12
N161	X	13	12	14	12	13	12	13	14	7	13	14	14	15	11	11	15	9	17	18	10	15
	Y	13	13	15	18	13	12	15	16.2	11	14	15	16	15	12	14	15	9	12	16	13	12
N162	X	12	8	14	12	13	11	11	14	7	10	14	15	15	11	10	15	8	14	18	12	14
	Y	15	14	15	17	14	12	11	15	7	12	14	16	16	12	11	16	11	13	15	14	11
N163	X	13	13	13	12	14	12	10	13	11	13	14	14	11	9	12	13	10	14	20	10	14
	Y	14	13	14	12	14	13	13	15.2	12	14	15	15	17	12	14	15	11	10	16	15	13
N164	X	12	13	14	16	14	11	12	13	7	12	14	13	15	9	9.1	13	10	14	18	10	17
	Y	16	14	15	16	14	13	14	15.2	13	13	15	14	16	9	11	15	12	13	17	13	12
N165	X	14	11	15	12	11	12	11	12	12	13	14	13	15	11	14	16	9	14	17	10	14
	Y	15	12	15	17	14	13	11	13	12	13	15	15	16	12	15	16	10	12	15	13	12
N166	X	14	10	14	17	13	12	11	13	7	13	13	13	15	11	11	13	9	18	20	10	14
	Y	15	11	14	17	14	14	13	14	12	14	14	14	16	12	11	15	11	12	16	13	11
N167	X	12	13	14	12	11	12	11	14.2	11	13	14	13	11	9	11	14	8	14	17	10	16
	Y	16	13	16	17	13	13	13	15.2	12	13	15	13	15	12	14	15	9	12	16	15	11

N168	X	14	11	13	14	11	12	14	13	7	12	14	12	11	11	14	16	9	14	17	10	14
	Y	14	13	13	16	13	12	14	16.2	7	13	14	14	16	11	14	16	9	12	15	12	12
N169	X	14	12	13	12	14	12	11	13	7	13	15	13	15	11	11	14	9	14	18	12	14
	Y	14	12	14	17	14	13	15	15.2	12	14	16	14	16	12	12	14	10	13	15	12	12
N170	X	12	12	14	16	13	13	11	13	7	14	13	13	11	10	11	13	9	14	17	8	14
	Y	14	12	17	16	14	13	14	15.2	7	14	15	13	17	12	12	16	10	12	14	13	11
N171	X	15	12	13	12	11	11	14	14.2	7	12	14	14	15	9	11	15	10	17	18	10	15
	Y	16	12	14	16	11	12	15	16.2	11	14	14	15	16	12	13	15	10	10	15	14	12
N172	X	14	12	13	12	11	13	11	13	7	13	13	13	11	11	10	13	10	14	17	8	14
	Y	16	13	15	18	14	14	13	13	11	13	14	15	17	12	11.3	15	11	11	16	12	12
N173	X	13	11	15	12	11	12	11	13	7	13	15	12	11	9	10	14	10	14	14	10	15
	Y	15	13	17	17	14	13	11	13	12	14	15	13	16	11	10	17	12	13	16	13	12
N174	X	13	12	13	12	13	10	11	13	7	13	14	14	15	12	11	15	8	18	18	12	14
	Y	14	13	13	12	13	11	11	15	12	13	14	15	16	14	12	15	10	13	15	13	12
N175	X	12	12	15	16	14	9	13	14	11	13	14	14	14	11	11	15	10	17	18	10	16
	Y	14	13	17	17	14	12	13	16	12	13	16	14	15	12	12	16	10	13	17	13	13
N176	X	13	11	14	17	11	9	11	13	12	10	14	13	11	12	11	15	9	14	20	10	15
	Y	14	13	15	17	14	12	14	14	12	15	15	15	17	13	12	16	10	12	15	12	12
N177	X	13	11	13	12	11	12	12	14	7	13	14	13	15	9	11	15	10	14	20	10	14
	Y	16	13	14	16	12	14	13	15.2	11	14	15	14	17	12	11.3	16	11	12	15	11	10
N178	X	14	12	14	16	13	12	14	14.2	11	13	13	10	15	11	10	14	9	14	17	10	15
	Y	14	13	15	17	14	13	15	16.2	12	13	15	15	17	12	12	14	11	12	16	13	13
N179	X	13	11	13	12	13	12	11	15.2	7	12	14	11	15	12	12	13	10	17	17	12	14
	Y	16	13	14	16	14	13	13	15.2	12	12	14	14	17	12	12	15	10	13	14	13	12
N180	X	14	11	15	16	10	13	11	13	7	13	15	14	14	11	11	13	10	14	18	10	15
	Y	14	12	16	19	14	13	14	13.2	11	13	15	16	17	12	11	15	10	12	16	11	11

N181	X	14	12	11	12	10	12	13	14	7	10	14	13	11	11	11.3	13	9	14	14	10	14
	Y	15	14	15	17	14	13	13	15	13	14	14	14	15	12	14	16	9	10	14	13	12
N182	X	14	11	12	16	11	10	11	13	11	12	15	14	13	11	11	14	8	17	18	10	14
	Y	15	14	14	17	14	11	13	14.2	12	14	15	16	16	13	14	15	8	13	15	13	13
N183	X	13	12	13	12	11	12	11	13	7	13	14	13	11	9	12	13	8	14	14	10	14
	Y	16	13	14	17	14	12	12	13.2	7	14	14	16	15	13	14	15	9	12	16	13	13
N184	X	13	11	13	12	14	12	11	14	12	12	14	14	11	9	10	15	8	14	18	12	14
	Y	15	13	14	17	14	12	14	14.2	12	13	14	14	17	12	10	15	9	13	15	13	12
N185	X	13	12	14	12	13	12	11	14.2	7	13	13	14	16	12	11	14	8	17	18	8	15
	Y	14	13	14	12	14	14	13	15.2	7	13	15	16	17	12	14	16	10	8	16	14	13
N186	X	13	12	13	12	13	12	11	13	7	13	14	12	11	11	11	14	9	14	18	8	14
	Y	15	13	16	16	14	13	14	14	12	14	14	12	16	13	11.3	15	11	10	15	13	11
N187	X	13	12	15	17	13	11	11	14	11	13	13	13	15	12	11	14	11	18	19	12	15
	Y	15	14	16	17	14	12	11	15	11	13	15	13	16	12	11.3	14	11	12	17	14	10
N188	X	13	12	14	12	13	12	13	14	7	10	13	13	11	7	10	15	10	14	14	12	15
	Y	15	13	16	16	13	12	14	14.2	12	13	14	14	15	10	14	15	12	13	15	14	13
N189	X	15	12	13	16	11	13	13	13.2	11	14	14	13	15	12	10	14	9	14	14	12	15
	Y	16	13	14	17	11	13	14	14	12	14	15	15	16	13	11	16	10	13	17	12	12
N190	X	13	11	14	12	11	9	11	14	7	14	14	13	16	11	11	15	9	17	17	10	15
	Y	13	13	15	12	15	10	11	15.2	13	14	15	13	16	12	11	16	11	12	17	14	13
N191	X	13	13	14	12	10	11	14	14	7	13	14	14	11	11	11	15	8	14	14	10	14
	Y	13	14	15	18	13	12	15	14.2	11	14	15	15	15	12	14	15	10	13	15	11	11
N192	X	16	13	14	12	11	11	11	15.2	7	13	13	12	11	9	11	14	9	14	17	10	15
	Y	16	15	15	17	14	12	13	15.2	12	14	16	16	17	14	12	16	11	12	15	13	13
N193	X	13	12	15	12	11	13	11	12.2	7	13	14	14	11	11	11	15	10	14	14	10	15
	Y	15	12	15	17	14	13	11	13	7	14	16	14	16	12	12	16	10	12	16	11	11

N194	X	13	12	13	12	14	11	13	13	7	10	14	13	15	9	10	15	9	17	18	10	15
	Y	15	13	15	17	14	11	15	15.2	7	13	16	13	16	11	11.3	16	10	11	17	13	12
N195	X	14	12	15	16	11	11	13	13	7	13	14	13	16	11	12	13	9	18	18	10	14
	Y	16	13	15	19	14	13	13	13	12	14	14	14	17	12	12	15	9	12	15	13	12
N196	X	14	13	12	12	10	8	11	14	7	10	14	13	11	11	10	13	10	14	14	10	14
	Y	14	13	15	17	13	13	14	14	12	14	14	15	15	12	11.3	15	10	11	15	13	13
N197	X	12	11	14	12	11	10	12	12	12	13	14	13	11	9	10	14	8	14	14	10	15
	Y	13	13	16	16	14	12	13	14	12	13	15	15	17	11	12	15	10	13	18	11	11
N198	X	13	11	14	17	12	12	11	12	7	13	14	13	11	9	13	14	9	14	17	12	14
	Y	14	12	15	17	14	12	14	13	12	14	16	13	15	14	13	15	10	12	14	12	12
N199	X	13	14	13	12	10	12	13	13	7	13	13	12	11	10	10	13	8	17	17	10	15
	Y	13	14	14	16	14	12	14	13.2	11	13	14	14	17	12	14	14	11	10	17	13	12
N200	X	12	8	14	17	11	13	13	13	7	12	14	14	11	12	10	14	9	18	20	10	15
	Y	13	12	14	18	14	14	14	14	12	14	15	15	15	12	11	14	10	10	15	14	12
N201	X	15	11	13	17	14	12	13	15	11	13	14	12	11	12	10	14	9	14	18	10	14
	Y	15	15	14	17	14	13	15	16.2	12	13	14	13	16	14	11	15	10	10	15	12	11
N202	X	12	12	14	16	11	12	13	12	7	13	14	10	11	9	11	15	10	14	17	8	14
	Y	14	12	15	16	14	12	13	13	11	13	16	13	16	11	13	15	11	10	15	13	12
N203	X	12	12	9	16	11	12	11	13	12	13	14	12	15	13	11	13	9	14	17	10	17
	Y	14	14	14	17	14	12	14	14.2	12	13	15	14	16	13	11	14	10	12	18	13	13
N204	X	14	11	15	17	13	11	12	12	7	12	12	14	16	12	10	15	9	14	14	8	14
	Y	14	13	15	17	14	13	14	13	7	14	13	15	16	13	10	15	10	13	14	13	11
N205	X	13	14	13	12	10	10	11	14	7	13	14	14	15	10	11	13	8	14	18	12	14
	Y	15	14	14	16	14	11	13	14.2	7	13	15	14	17	13	14	14	10	13	15	14	13
N206	X	15	13	15	17	13	10	11	13	7	13	13	14	11	12	10	14	10	18	18	10	17
	Y	16	13	15	17	14	12	14	14	7	14	15	15	16	12	11	16	11	13	17	13	13

N207	X	13	10	14	12	12	8	11	15.2	7	14	14	15	11	8	10	14	8	14	17	10	15
	Y	15	13	16	17	14	12	12	15.2	13	15	15	15	17	11	11	15	9	12	15	12	11
N208	X	13	12	13	17	12	8	13	13	7	13	13	11	11	12	13	13	8	14	17	8	15
	Y	13	12	15	17	12	12	14	14	12	14	14	14	16	15	14	15	10	12	15	12	11
N209	X	13	12	13	12	10	12	14	13	7	13	13	14	15	11	10	13	8	14	20	8	15
	Y	13	14	15	17	12	12	14	13	13	14	14	15	16	12	14	16	10	8	15	12	12
N210	X	15	11	13	12	14	12	11	13	7	13	12	14	16	11	11	16	10	14	18	10	15
	Y	15	12	14	12	14	12	12	14.2	7	14	15	15	17	12	11	16	10	10	15	13	12
N211	X	13	12	15	16	13	9	11	14	12	13	12	13	11	11	11	13	9	14	20	12	14
	Y	15	14	16	17	13	12	11	14	13	13	15	15	16	14	11.3	13	10	12	16	13	12
N212	X	15	10	15	14	14	12	11	13.2	7	14	13	13	11	9	10	13	9	14	18	10	14
	Y	16	13	16	18	14	12	11	13.2	12	14	15	14	16	9	14	15	10	12	14	14	14
N213	X	13	12	15	16	10	11	11	15.2	7	13	13	13	15	12	11	15	8	14	17	10	14
	Y	16	13	16	18	13	12	11	15.2	13	14	13	13	15	13	11	15	11	13	16	13	11
N214	X	12	12	16	16	11	11	11	13	12	10	13	13	14	11	10	13	8	17	17	8	15
	Y	13	13	16	18	14	11	13	15.2	12	13	14	14	17	11	11.3	14	11	10	16	13	11
N215	X	15	13	15	12	11	12	12	13	7	13	14	13	14	11	10	14	8	17	17	10	15
	Y	16	13	15	16	14	12	13	13	7	14	14	14	15	11	12	14	12	10	16	13	11
N216	X	13	12	13	12	10	12	11	13	7	13	14	13	15	12	12	15	9	14	23	8	15
	Y	15	14	14	16	13	13	11	13	7	13	14	14	17	12	12	17	10	12	15	13	13
N217	X	13	9	12	17	11	12	11	14	7	12	13	14	11	12	12	13	9	20	20	8	15
	Y	15	12	16	18	14	13	11	14.2	12	13	15	15	16	12	14	15	10	13	15	13	12
N218	X	14	11	12	16	11	13	11	12	7	13	13	13	11	12	12	13	8	20	20	8	15
	Y	15	12	15	17	14	14	11	14	12	13	14	14	16	12	14	16	10	12	16	13	12
N219	X	13	12	14	14	11	12	13	13	7	12	13	14	15	10	10	15	9	17	18	10	15
	Y	13	13	16	18	12	13	14	13	7	13	14	14	16	12	12	15	10	10	17	13	10

N220	X	13	11	14	17	12	12	13	13	7	13	13	13	15	10	11	13	9	14	18	10	14
	Y	14	12	14	18	14	13	14	14	7	13	14	14	17	12	12	15	9	10	17	10	10
N221	X	15	10	12	16	13	11	14	12	7	13	14	14	11	11	10	12	10	14	20	8	14
	Y	15	12	15	17	14	13	14	14	12	13	15	14	15	11	12	13	10	10	16	13	12
N222	X	12	8	15	12	11	12	14	14	11	13	11	13	16	12	11	15	8	14	14	9	15
	Y	13	14	16	17	13	12	14	14.2	12	13	14	14	17	12	12	16	10	10	17	12	12
N223	X	14	12	13	12	14	12	11	13	12	12	14	12	16	10	10	13	8	14	18	10	14
	Y	15	12	14	14	14	13	14	15.2	12	13	15	15	16	14	10	15	9	10	15	13	12
N224	X	14	11	14	16	11	13	11	14	7	13	15	13	11	11	10	15	8	14	17	10	14
	Y	14	13	15	17	14	14	11	14.2	12	13	17	14	11	12	12	15	10	10	15	14	13
N225	X	13	11	14	12	14	11	11	13	11	13	14	12	15	9	11	14	8	17	17	10	14
	Y	16	12	15	17	14	12	12	13	12	14	15	13	16	12	14	16	10	10	14	14	12
N226	X	12	11	13	12	11	10	11	14	7	13	14	13	11	12	10	13	9	14	23	8	14
	Y	17	13	14	16	13	12	11	15.2	11	13	14	14	15	12	11	13	11	10	16	13	12
N227	X	14	11	12	16	13	13	11	13	7	13	14	12	16	9	12	15	10	14	20	8	14
	Y	15	12	14	17	14	15	14	14	7	13	16	16	17	12	12	16	11	13	15	12	11
N228	X	13	12	13	12	13	12	13	14	7	11	14	13	11	12	10	13	11	14	17	13	14
	Y	15	13	14	16	14	12	13	15	12	14	14	14	17	14	12	15	11	13	15	14	13
N229	X	14	11	15	17	13	9	13	14	7	13	12	15	11	9	11	15	10	14	14	8	15
	Y	14	12	16	18	14	13	14	14	7	14	15	15	17	14	11	16	11	12	15	14	14
N230	X	13	11	13	12	13	12	11	12	7	14	14	13	15	11	10	13	8	14	18	12	15
	Y	15	12	13	16	14	13	15	15.2	12	14	15	16	17	12	11	15	9	13	15	13	12
N231	X	13	12	12	16	14	9	10	13	7	14	14	13	11	12	9.1	13	9	17	17	8	14
	Y	16	14	15	16	14	12	13	15.2	12	15	14	15	16	12	12	14	9	10	16	12	12
N232	X	13	13	15	16	14	10	11	14.2	7	13	14	14	15	10	9.1	13	8	14	14	10	14
	Y	13	14	16	17	14	13	11	15.2	7	14	14	14	18	13	10	14	10	12	17	11	10

N233	X	13	12	14	16	12	13	12	14	7	13	14	14	16	11	11	15	9	14	17	8	14
	Y	14	14	14	17	14	14	14	15.2	12	14	15	14	17	12	14	15	10	10	15	13	11
N234	X	13	11	15	12	13	12	11	14	7	12	13	13	16	9	11.3	14	8	14	14	8	14
	Y	16	11	18	12	13	13	13	14.2	7	13	15	14	17	12	12	15	8	10	17	14	12
N235	X	13	11	12	12	13	11	13	12	11	13	14	13	16	8	12	16	11	14	18	10	14
	Y	14	12	13	17	14	12	14	15.2	12	13	16	14	16	11	12	16	11	10	17	13	11
N236	X	12	10	14	12	10	11	12	14.2	7	13	15	14	15	11	10	16	9	17	20	8	16
	Y	14	12	15	16	14	12	14	15.2	7	14	16	15	17	12	14	16	11	10	17	13	11
N237	X	13	12	15	12	13	8	13	13	7	13	15	14	11	10	10	15	9	17	18	10	14
	Y	13	14	15	17	14	12	13	14	11	13	17	14	17	11	11.3	16	12	13	15	12	10
N238	X	12	12	13	12	11	12	11	13	11	10	13	13	11	11	11	15	9	14	18	8	14
	Y	15	12	15	12	15	12	12	14	11	13	14	13	18	12	12	16	9	10	15	13	12
N239	X	13	13	15	12	11	12	11	14	7	13	14	14	15	13	11	14	9	14	17	10	16
	Y	15	17	16	16	14	13	14	15.2	11	14	14	15	17	13	11	16	10	11	17	13	13
N240	X	14	12	13	16	14	12	11	14	11	12	12	14	16	9	12	14	10	17	18	10	15
	Y	15	12	15	17	14	12	11	15.2	12	13	15	14	16	11	12	14	10	13	15	14	13
N241	X	13	13	14	12	9	12	11	13	7	13	14	13	16	12	11	13	9	14	18	10	14
	Y	15	13	14	16	10	12	11	14	7	13	14	14	17	12	11	13	10	13	15	11	10
N242	X	12	11	13	12	11	12	11	15.2	11	10	15	12	15	11	11.3	13	9	17	18	10	14
	Y	15	12	14	17	14	14	13	15.2	12	12	16	15	17	12	12	15	9	12	15	13	11
N243	X	13	12	14	12	13	12	11	14.2	7	13	13	14	11	11	11	13	8	14	17	12	15
	Y	15	13	16	16	14	12	13	15	12	13	14	15	17	12	14	16	9	12	15	12	12
N244	X	13	14	15	17	14	12	12	14	11	13	14	15	16	10	11	14	9	14	14	10	15
	Y	14	14	16	17	14	13	15	14	12	14	15	16	16	10	14	15	11	10	15	15	12
N245	X	13	10	14	17	11	12	14	13	11	13	13	13	11	12	10	14	8	14	20	12	15
	Y	13	11	15	17	14	13	15	14	12	13	14	13	15	12	10	15	11	13	18	13	12

N246	X	12	12	15	12	13	11	14	13	7	13	14	13	16	9	10	14	8	14	18	8	14
	Y	13	15	16	17	14	12	14	16.2	13	13	15	14	16	11	11	14	9	10	14	12	11
N247	X	13	12	12	12	14	12	13	13	7	13	14	15	11	11	10	16	8	14	14	10	14
	Y	13	12	15	18	14	12	15	14	12	14	14	15	16	12	14	16	10	12	15	13	11
N248	X	13	11	13	12	13	12	11	13	7	14	14	13	16	13	10	13	8	14	17	11	14
	Y	15	11	15	16	14	12	13	14.2	7	14	14	14	17	13	12	15	9	12	16	13	11
N249	X	12	12	13	12	12	12	11	13	11	13	13	13	15	9	11	14	10	14	17	8	14
	Y	13	13	13	14	13	14	13	13	12	13	16	14	15	11	11	15	10	10	15	13	13
N250	X	13	12	13	12	10	12	10	14	7	13	14	13	11	9	9.1	14	8	14	18	8	14
	Y	15	13	14	17	14	13	14	17.2	11	14	14	13	15	11	14	14	8	10	15	13	12
N251	X	13	12	14	14	13	10	11	13	7	12	13	15	15	11	9.1	15	10	14	17	8	15
	Y	15	13	15	17	14	10	14	14	7	13	15	15	17	12	14	16	12	12	15	12	12
N252	X	13	12	14	12	11	11	11	13	7	14	15	14	11	9	10	13	8	14	14	10	17
	Y	15	15	15	16	14	13	12	16.2	7	14	15	16	17	11	11	14	10	13	17	11	11
N253	X	13	12	14	16	13	12	13	13	7	13	15	13	11	12	12	15	10	14	18	8	14
	Y	14	14	15	17	13	12	14	15	11	13	15	13	15	13	14	16	10	12	15	13	11
N254	X	13	12	14	12	14	12	13	13	7	13	15	10	11	11	10	13	10	14	20	10	15
	Y	14	13	15	14	14	12	15	15.2	7	13	15	14	17	12	11	13	10	10	15	14	13
N255	X	13	11	13	12	13	12	11	14.2	7	13	13	14	16	10	10	13	10	14	17	12	15
	Y	13	13	15	16	14	12	14	15	11	14	14	15	16	12	12	15	10	13	15	13	12
N256	X	15	11	13	12	13	12	11	14	7	13	14	13	14	11	10	13	11	14	18	10	14
	Y	16	12	15	17	14	13	12	14	12	13	15	15	15	12	11	16	11	10	15	12	12
N257	X	14	12	12	12	10	11	14	13	7	13	14	14	16	12	11	13	9	17	17	10	15
	Y	18	13	13	17	14	13	14	15.2	7	14	15	14	17	13	15	15	10	10	16	13	11
N258	X	12	12	14	12	14	12	11	13	12	13	14	13	15	9	10	15	9	14	14	10	14
	Y	15	12	15	16	14	14	11	14.2	12	13	14	13	16	12	12	15	9	11	15	13	11

N259	X	15	11	14	12	11	11	11	14	7	13	13	13	11	11	13	15	9	14	17	10	15
	Y	16	13	14	12	14	13	12	14.2	11	14	15	14	15	12	14	16	11	10	16	13	10
N260	X	12	12	13	16	14	10	11	14	7	13	13	13	17	11	14	14	9	17	17	10	14
	Y	15	13	14	18	15	11	12	14.2	12	14	13	14	17	11	14	15	9	12	14	13	11
N261	X	14	12	13	17	14	12	11	12	7	10	13	13	15	11	10	13	10	17	17	10	17
	Y	14	13	14	18	14	12	13	13.2	12	13	15	13	17	12	14	17	11	13	17	13	12
N262	X	13	12	14	12	10	12	13	13	7	13	15	15	17	9	11	14	8	14	17	12	14
	Y	13	13	15	12	11	13	14	14	7	14	15	15	17	12	11	16	8	14	15	12	11
N263	X	14	11	14	12	10	11	11	13	7	13	13	14	11	11	9.1	14	9	18	18	10	15
	Y	15	13	15	17	14	12	14	14	12	13	15	16	17	12	10	16	10	13	15	13	12
N264	X	13	13	14	17	10	11	13	14	7	13	13	13	16	11	12	14	8	14	14	10	14
	Y	15	14	14	17	14	12	13	15	7	13	15	14	17	13	12	15	10	12	15	13	13
N265	X	14	12	15	12	13	12	11	14.2	7	12	14	14	15	9	11	14	8	14	17	8	14
	Y	15	12	16	17	14	14	11	15.2	11	13	16	14	17	11	11	15	10	10	17	12	12
N266	X	13	11	15	12	11	12	11	14.2	7	12	16	14	15	9	12	13	8	17	18	8	14
	Y	13	12	16	12	14	13	11	15.2	12	13	16	14	17	12	14	14	10	10	15	13	11
N267	X	13	12	13	17	13	11	11	14	11	12	12	14	11	9	12	14	9	14	14	10	15
	Y	14	12	16	17	14	11	14	14	12	12	14	15	17	10	14	15	10	11	16	11	11
N268	X	13	11	14	16	11	11	13	12	7	13	14	13	15	9	10	15	9	14	18	8	15
	Y	16	12	15	17	12	12	14	13.2	13	14	15	14	17	12	14	15	11	10	16	12	12
N269	X	15	12	14	12	12	12	11	15.2	7	12	15	13	16	10	11	15	9	14	17	10	14
	Y	16	14	16	17	14	13	11	16.2	13	13	15	14	17	14	11	15	10	12	15	13	13
N270	X	14	14	13	12	11	11	14	14	7	10	15	13	15	11	10	13	8	14	17	8	15
	Y	14	14	16	14	13	13	14	14.2	11	13	15	13	16	11	11	16	10	13	15	12	12
N271	X	13	14	14	17	13	12	11	13	12	12	14	12	17	12	12	14	8	17	18	10	14
	Y	14	14	14	18	14	15	11	14.2	12	12	14	14	17	14	14	15	11	10	16	14	11

N272	X	13	13	14	12	14	12	11	13	11	13	16	14	15	11	11	13	8	14	17	10	15
	Y	15	14	17	16	14	12	13	15.2	12	14	17	15	17	12	11	17	9	13	17	13	12
N273	X	13	11	12	14	14	12	11	13	7	12	15	14	11	12	11	13	10	18	20	8	15
	Y	15	14	15	19	14	13	13	14	12	14	15	15	17	13	11.3	14	10	12	15	15	11
N274	X	14	13	15	12	13	11	13	13	7	12	14	14	15	11	10	15	9	14	17	10	15
	Y	15	13	16	12	13	12	14	14	7	14	14	14	16	12	11	16	10	13	15	14	14
N275	X	12	12	15	12	11	11	11	14	12	12	14	15	15	9	11	13	8	14	17	12	15
	Y	13	12	16	16	13	12	13	15.2	12	13	15	16	17	12	11	16	9	12	17	12	11
N276	X	15	11	13	12	13	12	11	14.2	7	10	13	13	15	12	10	13	10	14	14	10	15
	Y	15	12	14	12	14	12	11	16.2	12	13	13	14	16	12	13	15	12	10	15	13	10
N277	X	15	12	14	17	13	11	11	13.2	7	14	14	14	16	12	11.3	14	9	17	18	8	14
	Y	15	13	15	17	14	12	12	15.2	12	14	16	14	17	14	14	15	11	10	15	14	12
N278	X	16	8	13	12	13	10	11	13	7	12	12	13	17	9	11.3	13	10	14	17	10	14
	Y	17	11	14	16	14	14	11	16.2	12	14	14	15	17	13	11.3	15	11	13	16	13	11
N279	X	14	12	13	12	13	11	14	13	7	12	14	13	11	11	11	13	10	17	17	8	14
	Y	16	13	15	12	14	12	14	13	7	14	15	14	16	13	16	14	12	10	15	13	11
N280	X	14	12	13	12	11	11	11	13	7	13	13	14	15	11	11	13	9	14	17	8	14
	Y	15	14	14	13	13	12	11	14	11	15	16	16	17	11	13	13	11	13	15	13	11
N281	X	13	12	14	12	11	9	11	14.2	7	13	14	15	15	11	11	14	9	18	18	8	15
	Y	14	13	14	16	14	12	11	15.2	11	14	15	15	16	13	15	15	11	12	17	13	11
N282	X	13	13	13	16	13	11	11	14	7	13	14	14	15	11	11	14	8	14	14	8	15
	Y	15	13	14	16	13	12	13	14	12	13	15	14	15	12	11	15	9	12	17	15	12
N283	X	14	13	15	12	14	12	13	14	11	13	15	13	16	11	11	15	10	14	14	8	14
	Y	17	14	16	16	15	13	15	16	11	14	16	14	17	12	12	16	11	10	16	13	12
N284	X	12	11	14	12	14	12	11	13	7	13	15	12	15	12	10	13	8	14	17	8	14
	Y	15	11	15	16	14	14	14	14	13	13	15	13	16	12	11	15	8	8	15	13	12

N285	X	13	12	14	12	12	12	11	13	11	12	14	14	16	10	10	13	8	14	18	10	14
	Y	14	13	15	16	14	12	11	16	11	13	15	15	16	12	14	17	9	12	16	13	13
N286	X	12	11	13	12	13	10	11	13	7	13	13	13	15	12	10.1	13	9	17	17	9	15
	Y	12	12	14	18	14	12	11	14.2	11	14	15	15	17	12	11	16	10	12	17	16	12
N287	X	13	11	13	14	11	11	11	13	7	13	15	14	16	10	10	14	9	18	18	10	15
	Y	15	12	15	17	13	11	11	14	11	13	16	14	16	12	11	15	10	12	17	13	13
N288	X	13	13	14	12	13	12	11	14.2	7	12	14	14	15	11	14	13	10	18	18	10	15
	Y	13	14	16	17	13	13	14	15.2	11	13	14	15	16	11	14	15	10	11	17	13	11
N289	X	13	11	15	14	11	11	11	13	7	13	15	10	11	9	12	13	10	14	18	10	14
	Y	13	12	16	16	14	13	14	14.2	11	13	15	13	15	10	13	16	11	12	17	12	12
N290	X	12	11	13	17	13	10	14	13	7	13	13	10	11	12	11	13	9	14	14	10	15
	Y	15	13	15	17	14	12	14	14.2	12	14	18	14	15	13	12	16	10	12	16	13	10
N291	X	13	12	14	12	14	11	11	13	7	13	15	15	11	11	10	13	11	17	17	10	14
	Y	14	12	15	17	14	12	13	15	12	14	15	15	17	11	14	15	11	12	17	13	12
N292	X	13	13	12	16	14	12	11	14	7	13	13	13	15	12	13	13	8	18	23	9	14
	Y	15	14	14	19	14	12	14	14.2	12	13	15	14	15	12	14	15	10	12	15	13	11
N293	X	14	12	15	12	14	12	11	13	7	12	14	14	11	11	12	13	9	14	18	10	15
	Y	15	13	15	12	14	12	11	15	12	13	15	15	15	11	14	14	9	12	15	13	13
N294	X	12	14	14	12	12	10	11	13	11	13	14	12	15	9	11	14	8	14	20	10	14
	Y	14	14	15	17	14	13	13	15.2	12	13	14	14	15	14	11	16	9	12	15	13	12
N295	X	13	8	14	12	14	13	11	14	7	13	14	13	11	10	12	13	10	17	17	10	14
	Y	15	14	14	16	16	14	11	16	12	14	15	14	15	11	14	16	11	12	16	12	11
N296	X	13	13	14	12	10	11	11	13	12	14	13	14	15	8	11	13	8	14	14	11	14
	Y	15	13	16	16	14	12	14	14	12	14	13	15	15	13	11.3	13	8	13	16	14	12
N297	X	13	11	14	12	13	12	13	14	7	13	14	13	16	9	11	13	8	14	18	8	14
	Y	15	12	15	16	14	12	14	15	12	13	15	15	16	11	11	15	10	10	15	14	13

N298	X	13	12	14	12	11	10	13	13	7	13	14	13	15	13	11	13	8	14	18	10	14
	Y	14	13	14	16	13	11	14	15.2	12	14	15	15	16	13	14	14	9	12	14	13	12
N299	X	12	11	12	17	10	11	11	14	7	13	15	14	16	11	10	13	8	17	18	10	15
	Y	15	12	15	17	10	11	11	14	7	13	15	15	16	12	12	15	11	10	16	13	12
N300	X	11	12	15	17	14	12	14	14.2	7	13	15	13	13	10	11	14	8	14	17	8	15
	Y	12	12	15	18	14	12	14	15	12	14	16	14	17	12	14	14	9	12	16	13	12
N301	X	15	11	14	12	13	9	11	13	7	13	13	13	16	10	11	13	10	17	18	11	14
	Y	15	14	15	12	14	12	13	14	7	14	14	15	17	11	14	15	11	12	15	13	13
N302	X	15	12	13	12	11	13	13	13	7	13	14	12	11	11	10	13	9	14	14	8	14
	Y	15	13	14	12	11	13	15	15.2	12	13	15	13	11	12	10	15	9	13	16	13	13
N303	X	13	13	12	12	10	11	11	13	11	13	14	16	15	11	10	15	9	14	14	8	14
	Y	13	14	13	16	14	12	11	14	12	14	14	17	16	11	13	17	10	12	15	12	12
N304	X	13	11	13	16	13	12	10	14	7	13	14	14	11	9	11	13	10	17	20	10	14
	Y	14	14	14	16	14	12	11	16.2	12	13	15	14	16	11	11	16	10	10	17	13	13
N305	X	13	11	13	16	10	11	11	13	12	13	14	13	11	11	11	13	9	14	14	11	14
	Y	14	12	15	17	14	12	11	14	12	14	14	14	17	14	13	13	11	13	17	13	11
N306	X	13	12	14	12	13	11	10	14	7	13	14	13	17	12	10	13	9	18	20	10	15
	Y	14	13	16	18	13	12	11	15.2	11	14	14	14	18	12	11	15	9	13	15	13	13
N307	X	14	12	14	12	14	11	11	14.2	7	13	13	14	15	9	11	13	10	14	18	10	15
	Y	15	12	16	18	14	12	14	14.2	11	13	14	16	17	13	14	14	10	13	17	13	11
N308	X	13	11	14	14	13	12	11	12.1	7	13	14	14	16	11	10	13	9	14	18	10	14
	Y	16	12	15	18	15	13	13	14.2	12	13	15	14	17	12	10	14	10	12	15	12	12
N309	X	14	11	10	12	11	10	11	13	7	14	13	13	11	11	10	15	10	14	17	10	14
	Y	15	12	10	17	13	12	14	14	12	14	14	14	16	12	11	15	11	12	15	12	11
N310	X	14	12	14	17	13	12	12	14	7	13	13	14	15	11	11	15	8	17	18	10	14
	Y	14	14	15	17	14	13	14	14	7	14	16	14	17	12	14	16	8	10	15	13	12

N311	X	13	12	13	12	13	11	11	13	7	13	14	13	15	11	8	15	8	14	17	10	14
	Y	14	13	14	17	13	12	11	13	11	13	14	14	16	12	12	15	10	12	16	13	11
N312	X	13	12	15	12	13	10	12	13	11	12	14	13	15	12	11	13	8	14	17	8	14
	Y	13	12	16	12	14	12	14	14	12	14	16	14	17	12	12	17	10	10	18	12	11
N313	X	12	12	14	12	13	11	11	14.2	11	13	14	13	11	11	10	13	9	17	18	10	14
	Y	13	14	16	16	13	13	14	15.2	13	14	14	14	16	12	10	15	9	12	15	14	13
N314	X	13	12	14	12	10	13	13	14.2	7	13	14	13	11	12	10	14	10	17	17	8	14
	Y	14	13	15	16	15	13	15	14.2	12	13	15	14	15	12	14	16	11	10	14	12	11
N315	X	13	13	13	17	11	12	11	13	11	12	13	10	15	11	11	13	8	14	17	10	15
	Y	15	14	14	17	14	14	15	13	12	13	13	14	17	12	11	15	12	12	15	15	12
N316	X	13	12	14	17	11	10	13	13	7	13	13	13	11	12	12	13	8	18	20	10	14
	Y	14	12	14	18	14	11	14	14	12	14	14	16	16	13	14	17	9	10	14	12	10
N317	X	15	12	13	12	14	12	11	14	7	13	15	13	11	9	11	15	8	17	20	10	14
	Y	15	13	14	16	14	12	14	14	11	13	16	14	16	11	12	15	10	10	14	14	13
N318	X	12	12	14	12	11	12	11	13	7	13	14	12	11	11	10	13	8	14	20	12	14
	Y	12	13	16	12	13	12	14	15.2	7	13	14	14	15	12	14	15	11	13	16	14	13
N319	X	14	13	15	12	14	10	11	13	7	13	14	14	15	9	11	15	9	17	18	9	15
	Y	15	14	16	17	15	11	11	15.2	12	14	16	15	15	10	11	15	11	13	17	12	12
N320	X	13	12	14	17	10	11	13	13	7	14	14	10	17	12	10	14	9	14	18	8	15
	Y	15	13	15	17	14	13	15	15.2	11	14	15	13	17	12	11	15	10	12	15	13	11
N321	X	14	11	14	12	13	13	14	13	7	13	14	14	17	10	11	15	9	14	17	12	14
	Y	14	13	16	18	14	14	14	13.2	12	14	14	15	17	13	12	16	11	12	15	14	12
N322	X	14	11	14	12	13	9	11	14.2	11	12	14	13	15	12	12	15	8	14	18	8	15
	Y	15	12	16	17	14	11	14	14.2	12	13	14	15	17	12	13	15	10	10	15	13	11
N323	X	13	12	13	12	10	12	11	13	7	14	15	13	15	11	11.3	15	8	14	17	10	14
	Y	13	12	14	19	14	12	14	14.2	12	15	15	15	15	12	12.3	15	12	10	16	13	11

N324	X	13	11	14	16	14	10	11	13	12	13	14	14	17	9	12	14	9	14	14	10	16
	Y	14	13	15	17	14	12	11	14.2	12	13	14	14	18	14	12	16	10	11	17	13	12
N325	X	12	12	13	17	11	10	10	13	11	13	14	11	11	11	10	15	11	18	20	10	14
	Y	13	13	14	18	12	12	11	15.2	11	15	14	14	11	12	11	17	11	13	14	13	11
N326	X	12	12	13	14	14	9	13	13	12	14	13	13	15	11	10	15	8	17	17	8	14
	Y	13	12	15	16	14	12	14	15.2	12	14	14	13	17	12	12	16	10	12	14	13	13
N327	X	14	12	13	12	13	10	11	13	11	10	14	14	15	11	10	13	8	14	17	11	15
	Y	14	14	14	19	14	11	15	15.2	11	12	16	15	16	12	10	15	9	13	16	13	11
N328	X	14	11	14	12	13	12	11	14	7	14	15	13	16	11	10	14	9	18	18	8	14
	Y	15	12	15	17	14	13	12	14.2	11	14	15	14	16	14	14	16	11	8	17	13	13
N329	X	13	12	14	16	10	11	14	12	7	13	14	14	15	11	10	13	10	14	18	8	14
	Y	13	13	15	16	14	12	15	14.2	7	13	14	14	17	12	10	15	12	10	15	12	11
N330	X	13	11	12	12	11	11	11	14	7	13	14	14	15	9	10	14	9	14	14	10	14
	Y	16	12	16	16	14	13	14	16.2	7	13	15	14	17	11	10	15	9	12	15	12	12
N331	X	12	11	15	12	13	13	11	13	7	13	15	14	15	12	11.3	13	10	14	14	8	15
	Y	13	12	15	17	14	13	14	15	13	14	16	14	17	14	14	15	11	10	16	13	13
N332	X	14	12	12	12	12	12	12	13	7	13	14	13	17	9	10	16	9	14	17	8	15
	Y	14	13	14	17	15	12	15	14.2	7	13	14	14	18	13	14	16	9	10	15	13	10
N333	X	13	12	13	12	11	12	11	14	7	14	14	12	16	11	10	15	9	17	20	10	14
	Y	16	14	15	18	13	13	13	16	12	14	14	12	16	12	14	16	10	12	16	12	11
N334	X	12	12	15	16	10	12	11	13	7	13	13	13	11	12	11	15	10	14	17	10	14
	Y	16	14	15	17	13	13	13	15.2	11	13	14	15	17	12	14	16	10	11	16	13	12
N335	X	16	12	14	12	12	12	11	13.2	7	14	14	14	16	10	12	14	10	14	17	10	14
	Y	16	13	16	12	13	14	14	14	12	15	15	16	17	11	14	16	11	10	14	12	12
N336	X	13	12	13	12	13	11	13	14.2	11	13	14	14	11	9	11	16	8	14	18	12	14
	Y	14	13	14	12	14	12	15	15.2	11	13	15	15	15	11	13	17	8	12	17	13	12

N337	X	14	13	13	12	10	12	11	13	11	12	13	13	15	9	12	13	9	14	18	10	16
	Y	15	14	13	12	10	13	12	15.2	12	13	14	14	15	12	12	15	12	13	16	13	12
N338	X	16	11	15	17	10	12	13	14	12	13	13	13	11	13	11	15	10	17	18	8	14
	Y	16	12	15	18	14	13	14	14	12	14	14	15	17	13	11	16	10	10	14	12	11
N339	X	13	11	13	12	13	12	11	15.2	7	13	13	13	11	11	10	14	9	17	18	8	14
	Y	15	13	15	16	15	13	14	16.2	12	14	13	13	17	12	11	14	11	10	17	13	12
N340	X	14	11	12	16	14	12	11	15	7	13	14	15	15	9	12	13	10	18	20	11	14
	Y	15	12	15	19	15	13	11	15.2	7	14	15	16	15	12	14	14	11	12	14	12	10
N341	X	15	12	14	16	11	10	13	14.2	7	13	13	13	15	11	10	15	9	16	17	10	16
	Y	15	14	15	17	13	12	14	17.2	12	13	15	13	16	11	11	15	9	10	17	12	10
N342	X	13	12	13	17	14	10	11	13	7	13	14	13	11	11	10	14	10	18	20	8	14
	Y	15	13	15	18	14	12	11	15.2	7	14	16	13	15	12	12	14	11	10	16	13	12
N343	X	13	12	14	17	14	10	15	13	11	13	14	13	15	11	10	14	8	14	17	10	15
	Y	15	13	15	17	14	11	15	14	11	14	14	14	15	12	11	16	8	10	15	13	12
N344	X	13	11	13	14	13	12	13	13	12	12	13	15	11	11	11	13	9	17	23	12	14
	Y	14	12	14	16	14	13	14	14	12	13	15	16	15	11	12	17	9	13	16	13	13
N345	X	13	11	16	12	14	10	11	13	7	10	14	13	17	12	10	13	9	14	18	10	15
	Y	14	14	17	12	15	13	14	14	12	13	15	14	17	13	11	14	11	10	17	12	12
N346	X	13	12	13	16	13	10	14	14	7	13	15	13	11	9	11	13	9	14	18	8	14
	Y	16	14	14	17	14	13	14	14	7	13	15	14	15	9	13	14	10	12	14	13	12
N347	X	12	12	13	17	13	10	13	12	7	13	13	13	11	11	10	13	8	14	18	10	14
	Y	13	13	14	17	14	12	14	14	11	14	15	15	17	14	11	16	8	13	17	13	13
N348	X	13	13	15	16	11	12	14	12	7	13	14	14	14	11	10	13	9	17	20	12	14
	Y	13	13	16	17	14	12	14	13	12	14	15	15	15	13	14	15	11	13	14	12	11
N349	X	13	12	15	17	13	9	11	14.2	11	13	14	13	11	11	11	14	11	14	18	10	14
	Y	16	14	16	17	14	12	13	15	13	14	16	16	15	12	14	14	12	10	15	13	13

N350	X	13	11	14	12	10	12	13	14.2	11	13	14	13	15	11	10	15	10	14	18	8	14
	Y	14	12	15	14	11	12	14	16	11	13	15	15	16	12	11	15	10	13	15	13	11
N351	X	13	11	13	12	12	12	11	13	7	12	13	13	11	9	11.3	15	8	17	18	8	14
	Y	16	12	14	16	14	13	13	15.2	7	13	15	14	15	11	12	15	8	8	15	12	11
N352	X	15	11	13	17	13	12	10	15	11	13	13	13	16	9	10	15	10	18	20	12	14
	Y	15	12	15	18	15	14	11	15	11	13	15	14	16	12	11	16	11	13	15	13	12
N353	X	13	8	13	17	10	11	13	13	7	13	14	13	11	11	11	14	9	14	17	10	15
	Y	15	12	16	17	13	14	13	16.2	7	13	15	15	15	12	11	16	10	12	17	12	12
N354	X	14	11	13	12	11	13	11	15	11	13	14	13	15	11	11	15	9	14	20	8	15
	Y	17	13	14	16	12	13	13	15.2	12	14	14	13	17	12	11.3	16	10	12	16	14	12
N355	X	15	12	13	17	14	11	11	14	12	13	13	13	15	11	10	15	8	14	17	10	14
	Y	16	12	16	18	14	13	13	14.2	12	14	15	14	15	14	11.3	16	9	10	16	13	12
N356	X	13	11	12	12	14	12	14	13	7	11	14	14	15	9	10	14	9	14	17	13	15
	Y	14	14	15	17	15	12	15	14.2	11	13	15	14	15	12	11	17	10	13	16	13	10
N357	X	14	12	12	12	10	12	11	15	11	13	14	10	11	11	10	14	8	14	18	8	14
	Y	14	14	14	16	13	12	13	15.2	11	14	16	14	16	12	13	15	10	10	17	12	12
N358	X	14	13	12	12	11	12	14	13	12	13	14	14	15	11	11	15	10	17	18	10	14
	Y	15	13	14	16	14	13	15	16	13	15	14	15	15	12	12	16	10	12	16	12	12
N359	X	13	13	14	12	10	11	11	13	7	10	15	13	16	11	10	14	10	14	17	10	14
	Y	15	13	14	16	11	12	14	13.2	7	13	15	13	17	11	11	15	13	12	14	14	13
N360	X	13	12	13	16	13	12	10	13.2	7	13	15	14	11	11	11	14	9	14	14	10	14
	Y	13	13	14	17	14	12	14	15	12	16	16	14	15	12	14	15	10	10	14	14	13
N361	X	16	11	14	12	13	11	11	14.2	11	12	14	12	15	12	11	13	10	14	17	8	14
	Y	17	12	16	12	14	12	15	15.2	12	14	14	14	17	12	11	15	11	10	15	13	12
N362	X	13	12	15	12	13	11	13	13	12	13	14	13	15	11	10	14	9	14	20	10	14
	Y	13	14	17	17	13	12	13	14.2	12	13	15	14	16	12	11	15	11	12	15	13	12

N363	X	14	11	14	16	10	11	11	14	7	13	15	14	11	12	10	13	8	14	18	10	15
	Y	15	12	15	18	17	12	14	14.2	12	14	15	16	17	15	14	15	11	10	15	12	12
N364	X	13	8	12	16	13	10	11	13	7	13	14	14	15	11	11	14	11	17	18	12	14
	Y	15	13	15	17	14	13	14	15.2	12	13	15	14	17	12	14	16	11	13	15	13	12
N365	X	13	11	13	12	14	10	11	13	7	13	14	12	11	12	12	13	8	14	20	8	14
	Y	14	12	14	12	14	12	12	13	12	14	14	13	15	13	12	15	10	11	15	13	12
N366	X	13	13	14	16	13	11	11	13.2	7	12	14	10	15	9	12	15	10	14	17	8	15
	Y	15	14	15	18	13	13	14	15.2	11	13	15	13	16	11	12	16	10	10	16	14	12
N367	X	13	11	13	12	13	12	11	13.2	7	14	13	13	16	9	10	14	9	14	18	10	15
	Y	14	12	16	18	13	12	15	14	11	15	14	13	17	12	14	15	10	12	17	13	12
N368	X	13	11	15	14	14	13	11	14	11	10	14	12	15	11	10	15	8	14	17	8	15
	Y	14	11	15	17	15	13	11	15.2	12	14	15	13	16	12	11	16	9	10	15	13	12

Supplementary Table S3. The p value of the pairwise linkage disequilibrium in all pairs of 21 Non-CODIS STR loci in the Sichuan Han population.

Loci	[01]	[02]	[03]	[04]	[05]	[06]	[07]	[08]	[09]	[10]	[11]	[12]	[13]	[14]	[15]	[16]	[17]	[18]	[19]	[20]	[21]
[01] D10S1248																					
[02] D10S1435	0.1429																				
[03] D11S4463	0.8458	0.4314																			
[04] D12ATA63	0.6349	0.2708	0.5730																		
[05] D14S1434	0.3084	0.0773	0.2195	0.0963																	
[06] D17S1301	0.5480	0.3337	0.4941	0.5140	0.4044																
[07] D18S853	0.0160	0.7363	0.7625	0.6329	0.6431	0.3754															
[08] D19S433	0.0879	0.3577	0.6654	0.0082	0.2992	0.7977	0.2010														
[09] D1GATA113	0.0531	0.5653	0.2394	0.8619	0.5280	0.2003	0.0407	0.4491													
[10] D1S1627	0.9081	0.5303	0.0462	0.3359	0.8097	0.2630	0.2095	0.3747	0.9821												
[11] D1S1677	0.4370	0.2901	0.4255	0.0560	0.4616	0.0836	0.7868	0.7689	0.7128	0.8428											
[12] D20S482	0.8035	0.2020	0.5855	0.1713	0.1569	0.3317	0.9799	0.6492	0.3723	0.0065	0.0135										
[13] D22S1045	0.4929	0.3807	0.4496	0.4614	0.2231	0.9406	0.2570	0.7967	0.5614	0.8446	0.3490	0.9681									
[14] D2S1776	0.6928	0.0546	0.6974	0.3430	0.0162	0.6570	0.1760	0.8137	0.0051	0.3072	0.6699	0.0742	0.1349								
[15] D2S441	0.0837	0.6359	0.1945	0.2717	0.8655	0.4851	0.6963	0.0557	0.8668	0.3439	0.4494	0.1331	0.8366	0.5116							
[16] D3S4529	0.7476	0.6210	0.1468	0.1052	0.1355	0.8068	0.9263	0.1765	0.2484	0.2461	0.7445	0.6653	0.7469	0.4196	0.8449						
[17] D4S2408	0.0144	0.9967	0.7919	0.9807	0.4282	0.3833	0.9893	0.0298	0.8829	0.5088	0.0946	0.6500	0.1247	0.3305	0.6021	0.5035					
[18] D5S2500	0.2112	0.0510	0.9157	0.3155	0.5182	0.8235	0.4955	0.0080	0.7375	0.2835	0.3265	0.6545	0.1706	0.3763	0.3605	0.2850	0.3766				
[19] D6S1017	0.4512	0.6163	0.7147	0.5576	0.2442	0.3480	0.7291	0.3057	0.9533	0.2243	0.2626	0.6370	0.6380	0.8754	0.0602	0.8878	0.4590	0.6826			
[20] D6S474	0.3769	0.6289	0.3704	0.3143	0.2850	0.4039	0.9525	0.0994	0.2079	0.3648	0.8166	0.3710	0.1168	0.4688	0.8759	0.3182	0.6243	0.1392	0.8369		
[21] D9S1122	0.4650	0.7402	0.5719	0.0483	0.1116	0.6710	0.4545	0.5133	0.4373	0.3130	0.1274	0.7138	0.9188	0.0218	0.2822	0.6684	0.9962	0.0595	0.5701	0.3225	

Supplementary Table S4. Allelic frequencies of 21 STRs in Southwest Han population samples (n = 368).

Locus	Allele	Frequency	Locus	Allele	Frequency	Locus	Allele	Frequency
D10S1248	8	0.0014	D10S1435	8	0.0190	D11S4463	9	0.0027
	11	0.0054		9	0.0027		10	0.0027
	12	0.0788		10	0.0204		11	0.0027
	13	0.3682		11	0.1603		12	0.0503
	14	0.2351		12	0.3668		13	0.2024
	15	0.2174		13	0.2636		14	0.3098
	16	0.0815		14	0.1508		15	0.2785
	17	0.0109		15	0.0136		16	0.1332
	18	0.0014		16	0.0014		17	0.0163
D12ATA63	12	0.3505		17	0.0014		18	0.0014
	13	0.0014	D14S1434	9	0.0014	D17S1301	8	0.0054
	14	0.0340		10	0.0870		9	0.0272
	15	0.0014		11	0.1413		10	0.0666
	16	0.2052		12	0.0313		11	0.1698
	17	0.3057		13	0.2785		12	0.4524
	18	0.0897		14	0.4293		13	0.2269
	19	0.0122		15	0.0272		14	0.0435
D18S853	10	0.0177		16	0.0027		15	0.0082
	11	0.4063		17	0.0014	D1GATA113	7	0.4769
	12	0.0516	D19S433	12	0.0394		10	0.0014
	13	0.2283		12.1	0.0014		11	0.1766
	14	0.2296		12.2	0.0041		12	0.3098
	15	0.0652		13	0.3057		13	0.0353
	16	0.0014		13.2	0.0367	D22S1045	11	0.1984
D1S1627	10	0.0367		14	0.2364		13	0.0027
	11	0.0054		14.2	0.1168		14	0.0122
	12	0.1101		15	0.0611		15	0.2989
	13	0.5476		15.2	0.1481		16	0.2527
	14	0.2785		16	0.0149		17	0.2228
	15	0.0204		16.2	0.0326		18	0.0109
	16	0.0014		17.2	0.0027		19	0.0014
D1S1677	10	0.0014	D20S482	10	0.0245	D2S1776	7	0.0014
	11	0.0027		11	0.0082		8	0.0041
	12	0.0163		12	0.0571		9	0.1291
	13	0.1264		13	0.3179		10	0.0707
	14	0.4538		14	0.3709		10.1	0.0014
	15	0.3234		15	0.1671		11	0.3166
	16	0.0679		16	0.0530		12	0.3560
	17	0.0068		17	0.0014		13	0.0815
	18	0.0014	D3S4529	12	0.0014		14	0.0353
D2S441	8	0.0014		13	0.2283		15	0.0041

	9	0.0027		14	0.2337	D5S2500	14	0.4090
	9.1	0.0095		15	0.3329		16	0.0014
	10	0.2514		16	0.1671		17	0.2717
	10.1	0.0014		17	0.0353		18	0.2296
	11	0.3084		19	0.0014		19	0.0027
	11.3	0.0489	D4S2408	7	0.0014		20	0.0761
	12	0.1943		8	0.1943		21	0.0014
	12.3	0.0014		9	0.3016		23	0.0082
	13	0.0299		10	0.3342	D6S1017	7	0.0014
	14	0.1399		11	0.1345		8	0.1875
	15	0.0095		12	0.0326		9	0.0054
	16	0.0014		13	0.0014		10	0.4076
D6S474	12	0.0014	D9S1122	9	0.0014		11	0.0340
	14	0.3383		10	0.0435		12	0.2459
	15	0.3886		11	0.1576		13	0.1101
	16	0.1413		12	0.3315		14	0.0082
	17	0.1168		13	0.3859	D9S1122	15	0.0109
	18	0.0136		14	0.0666		16	0.0027

Fre.: Frequency.

Supplementary Table S5. The pairwise Fst and corresponding p-value of between the Sichuan Han population and 25 Chinese reference population. LST: Lhasa-Tibetan; QHS: Qinghai-Salar; HBTJ: Hubei-Tujia; YNB: Yunnan-Bai; NXH: Ningxia-Han; AU: Kuqa-Uyghur; ILK: Ili-Kazakh; FJS: Fujian-She; IMM: Inner-Mongolia-Mongolian; NH: Northern-Han; GNH: Guanzhong-Han; YNY: Yunnan-Yi; HNL: Hainan-Li; GGH: Guangdong-Han; HNH: Hunan-Han; HDH: Huadong-Han; BJH: Beijing-Han; ZJH: Zhejiang-Han; IMR: Inner-Mongolia-Russian; SDH: Shandong-Han; LNH: Liaoning-Han; HNH-2: Henan-Han-2; GSYG: Gansu-Yugu; XJXO: Xinjiang-Xibe; HNH-1: Henan-Han-1; SCH: Sichuan-Han.

Loci		LST	QHS	HBTJ	YNB	NXH	AU	ILK	FJS	IMM	NH	GNH	YNY	HNL
D1GATA113	Fst	-0.0040	-0.0034	-0.0005	-0.0016	0.0037	0.0042	0.0065	0.0098	-0.0012	-0.0004	-0.0014	0.0239	0.0000
	p	0.7452	0.7067	0.3947	0.4767	0.9862	0.0567	0.1090	0.0395	0.6271	0.4040	0.5822	0.0067	0.3615
D1S1627	Fst	0.0002	-0.0043	-0.0036	-0.0019	-0.0030	0.0023	0.0059	0.0165	-0.0010	-0.0033	-0.0026	-0.0044	0.0228
	p	0.3362	0.8838	0.7307	0.5100	0.8677	0.1191	0.1156	0.0091	0.5742	0.9656	0.8857	0.8604	0.0000
D1S1677	Fst	-0.0047	0.0102	-0.0046	-0.0012	0.0000	-0.0017	-0.0026	-0.0034	0.0120	-0.0020	0.0032	-0.0012	0.0020
	p	0.8719	0.0489	0.8667	0.4542	0.3697	0.8638	0.6464	0.8472	0.0008	0.6952	0.1158	0.4635	0.1353
D2S441	Fst	0.0021	-0.0020	-0.0012	-0.0010	0.0011	-0.0001	0.0057	-0.0013	0.0038	-0.0012	-0.0008	0.0023	0.0048
	p	0.2430	0.6279	0.5077	0.4920	0.2581	0.4186	0.0892	0.5654	0.0324	0.6197	0.5449	0.2195	0.0157
D2S1776	Fst	-0.0029	-0.0028	0.0002	-0.0029	-0.0032	-0.0003	-0.0033	-0.0017	-0.0015	0.0012	-0.0008	-0.0004	0.0044
	p	0.7001	0.7325	0.3668	0.7048	0.9740	0.4777	0.7883	0.6195	0.8485	0.2285	0.5433	0.4183	0.0318
D3S4529	Fst	0.0036	-0.0032	-0.0054	-0.0032	-0.0012	0.0026	-0.0040	0.0163	0.0001	0.0023	0.0032	-0.0035	0.0035
	p	0.1837	0.7685	0.9738	0.7190	0.5652	0.0906	0.8522	0.0024	0.3696	0.1780	0.1060	0.7707	0.0463
D4S2408	Fst	-0.0026	0.0021	-0.0005	0.0022	0.0000	0.0000	0.0151	0.0040	0.1119	0.0036	-0.0007	0.0012	0.0019
	p	0.6334	0.2419	0.4248	0.2501	0.3773	0.3841	0.0109	0.1205	0.0000	0.1030	0.4995	0.2989	0.1284
D5S2500	Fst	-0.0016	0.0053	-0.0044	-0.0035	-0.0031	-0.0014	0.0217	0.0066	0.0006	-0.0016	-0.0024	-0.0013	0.0009
	p	0.5138	0.1220	0.8421	0.7165	0.9101	0.7537	0.0038	0.0674	0.2800	0.6265	0.8637	0.4844	0.2392
D6S474	Fst	-0.0038	0.0070	-0.0037	-0.0005	0.0008	-0.0008	-0.0021	0.0042	-0.0001	-0.0002	-0.0009	-0.0010	-0.0003

	p	0.7644	0.0773	0.7656	0.4133	0.2861	0.5548	0.5848	0.1248	0.4088	0.4070	0.5410	0.4588	0.4395
D6S1017	Fst	0.0250	0.0033	-0.0051	-0.0018	0.0022	-0.0006	0.0001	0.0225	0.0076	-0.0014	0.0004	-0.0040	0.0092
	p	0.0013	0.1782	0.9580	0.5488	0.1809	0.5339	0.3715	0.0004	0.0055	0.6111	0.3292	0.8418	0.0026
D9S1122	Fst	-0.0038	-0.0034	-0.0046	-0.0046	0.0004	-0.0005	0.0031	0.0040	-0.0014	-0.0028	-0.0011	0.0027	-0.0016
	p	0.7876	0.7828	0.8909	0.8936	0.3180	0.4924	0.1901	0.1246	0.7808	0.9069	0.5902	0.2197	0.8595
D10S1248	Fst	-0.0015	-0.0019	-0.0048	-0.0050	-0.0026	0.0028	0.0008	0.0242	-0.0007	-0.0030	-0.0024	-0.0034	-0.0015
	p	0.5405	0.5951	0.9381	0.9457	0.8674	0.0722	0.3141	0.0001	0.5714	0.9472	0.8962	0.7713	0.8375
D10S1435	Fst	-0.0021	-0.0032	-0.0034	-0.0056	-0.0015	-0.0015	-0.0018	-0.0014	-0.0014	-0.0012	-0.0025	0.0168	-0.0002
	p	0.5886	0.7841	0.7803	0.9965	0.6459	0.8228	0.5849	0.5581	0.8231	0.5855	0.9417	0.0066	0.4391
D11S4463	Fst	-0.0029	0.0007	-0.0045	-0.0032	-0.0022	0.0041	0.0024	0.0098	-0.0020	-0.0021	-0.0021	0.0023	0.0009
	p	0.7040	0.3309	0.9186	0.7278	0.7713	0.0333	0.2250	0.0230	0.9705	0.7725	0.8469	0.2397	0.2300
D12ATA63	Fst	0.0397	0.0029	-0.0021	0.0009	-0.0006	0.0018	0.0232	-0.0008	0.0002	-0.0025	-0.0001	0.0039	0.0204
	p	0.0001	0.1944	0.5842	0.3136	0.4612	0.1367	0.0025	0.4807	0.3400	0.8526	0.3895	0.1572	0.0000
D14S1434	Fst	-0.0007	-0.0018	-0.0001	-0.0026	-0.0007	-0.0012	-0.0003	0.0046	-0.0001	-0.0015	0.0005	-0.0007	0.0014
	p	0.4401	0.5699	0.3858	0.6372	0.4649	0.7142	0.4116	0.0995	0.4035	0.6330	0.3051	0.4406	0.1716
D17S1301	Fst	0.0024	0.0007	-0.0020	-0.0018	-0.0022	0.0006	0.0103	0.0004	0.0000	-0.0026	-0.0028	0.0093	0.0030
	p	0.2200	0.3230	0.5761	0.5500	0.7627	0.2593	0.0344	0.3402	0.3798	0.8852	0.9857	0.0482	0.0699
D18S853	Fst	0.0024	-0.0033	-0.0042	0.0035	-0.0032	-0.0008	-0.0018	0.0016	-0.0005	-0.0019	-0.0029	-0.0026	0.0084
	p	0.2308	0.7772	0.8595	0.1872	0.9518	0.5956	0.5542	0.2530	0.5021	0.6975	0.9836	0.6612	0.0044
D19S433	Fst	0.0042	-0.0038	-0.0024	-0.0056	-0.0024	0.0053	0.0043	0.0081	-0.0015	-0.0021	-0.0021	-0.0012	-0.0005
	p	0.1316	0.9312	0.6952	0.9992	0.9078	0.0086	0.1070	0.0210	0.9176	0.8724	0.9150	0.5410	0.5444
D20S482	Fst	0.0040	-0.0027	0.0055	0.0068	-0.0016	-0.0002	0.0016	0.0316	0.0012	-0.0021	0.0020	-0.0042	-0.0015
	p	0.1569	0.6958	0.1154	0.0857	0.6364	0.4146	0.2612	0.0000	0.1907	0.7769	0.1656	0.8816	0.8116
D22S1045	Fst	0.0298	0.0594	0.0023	0.0027	0.0052	0.0017	0.0126	0.0866	0.0017	0.0325	0.0004	0.0851	0.0067
	p	0.0003	0.0000	0.2486	0.2176	0.0655	0.1466	0.0173	0.0000	0.1466	0.0000	0.3300	0.0000	0.0053

Supplementary Table S5. Continue

Loci		GGH	HNH	HDH	BJH	ZJH	IMR	SDH	LNH	HNH-2	GSYG	XJXO	HNH-1
	Fst	-0.0007	-0.0010	0.0000	0.0003	-0.0009	-0.0031	-0.0006	-0.0016	-0.0008	-0.0008	0.0001	-0.0032
D1GATA113	p	0.4838	0.5576	0.3608	0.3209	0.5507	0.6706	0.5183	0.5703	0.5700	0.4442	0.3442	0.9430
	Fst	0.0014	-0.0002	0.0019	-0.0023	-0.0009	0.0007	-0.0016	-0.0031	-0.0016	0.0067	0.0005	0.0064
D1S1627	p	0.1839	0.3889	0.2031	0.9946	0.5537	0.3136	0.9260	0.9137	0.9662	0.0730	0.2655	0.0587
	Fst	-0.0003	-0.0007	-0.0031	-0.0014	-0.0009	-0.0026	-0.0003	-0.0035	-0.0003	0.0065	-0.0012	-0.0024
D1S1677	p	0.4309	0.5045	0.9460	0.7029	0.5850	0.6298	0.4227	0.9799	0.4425	0.0557	0.7856	0.8051
	Fst	0.0001	-0.0012	0.0027	0.0012	-0.0017	0.0060	0.0010	-0.0025	0.0010	0.0013	0.0004	0.0087
D2S441	p	0.3837	0.7543	0.1265	0.1963	0.9094	0.0856	0.1762	0.8733	0.1768	0.2492	0.2923	0.0111
	Fst	0.0026	0.0018	0.0011	-0.0003	-0.0014	-0.0040	0.0009	-0.0023	0.0009	0.0018	-0.0009	-0.0016
D2S1776	p	0.0828	0.1340	0.2570	0.4504	0.8018	0.8828	0.1981	0.8070	0.1955	0.2119	0.7135	0.6824
	Fst	-0.0019	-0.0019	0.0024	0.0004	-0.0019	-0.0002	0.0022	0.0005	0.0027	0.0171	0.0010	-0.0005
D3S4529	p	0.9241	0.9171	0.1626	0.3078	0.9110	0.4024	0.0768	0.3317	0.0540	0.0016	0.1936	0.4725
	Fst	0.0001	-0.0020	-0.0032	-0.0019	-0.0008	-0.0037	-0.0001	0.0007	0.0001	0.0012	0.0003	0.0019
D4S2408	p	0.3646	0.9497	0.9744	0.9079	0.5880	0.8018	0.3953	0.3052	0.3729	0.2784	0.3198	0.1936
	Fst	-0.0009	-0.0014	-0.0035	-0.0022	-0.0011	-0.0029	-0.0010	-0.0027	-0.0014	0.0040	-0.0011	0.0075
D5S2500	p	0.5949	0.7481	0.9988	0.9683	0.6424	0.6658	0.7141	0.8219	0.8567	0.1257	0.7132	0.0334
	Fst	-0.0001	-0.0014	-0.0021	0.0003	-0.0002	0.0091	-0.0007	0.0003	-0.0008	0.0044	-0.0010	0.0051
D6S474	p	0.3857	0.7547	0.7384	0.3247	0.4188	0.0521	0.5986	0.3459	0.6293	0.1095	0.6892	0.0671
	Fst	0.0010	-0.0017	-0.0019	-0.0014	-0.0018	0.0005	0.0008	0.0036	0.0017	0.0110	0.0021	0.0013
D6S1017	p	0.2184	0.8651	0.7007	0.7578	0.8779	0.3454	0.2288	0.1136	0.1159	0.0134	0.0941	0.2439
	Fst	-0.0020	-0.0020	-0.0025	-0.0001	-0.0016	-0.0027	-0.0012	-0.0020	-0.0008	-0.0011	-0.0001	0.0049
D9S1122	p	0.9562	0.9552	0.8483	0.3929	0.8388	0.6668	0.8031	0.7065	0.6645	0.5087	0.4015	0.0635
D10S1248	Fst	-0.0004	-0.0009	-0.0035	0.0002	-0.0011	-0.0044	-0.0002	-0.0028	-0.0006	0.0016	-0.0009	-0.0020

	p	0.4989	0.6486	0.9999	0.3419	0.6941	0.9206	0.4379	0.9064	0.5905	0.2201	0.7064	0.7705
	Fst	-0.0013	-0.0014	-0.0026	-0.0012	0.0239	-0.0017	-0.0009	-0.0025	-0.0005	-0.0002	-0.0007	0.0012
D10S1435	p	0.7508	0.8205	0.9086	0.7050	0.0000	0.5757	0.7258	0.8477	0.5422	0.4173	0.6096	0.2499
	Fst	0.0014	0.0001	-0.0023	-0.0008	-0.0019	-0.0006	-0.0011	-0.0027	-0.0007	0.0057	-0.0008	-0.0006
D11S4463	p	0.1652	0.3794	0.8192	0.5743	0.9351	0.4480	0.8048	0.8911	0.6224	0.0618	0.6690	0.4858
	Fst	0.0029	-0.0008	-0.0022	-0.0003	-0.0016	0.0017	-0.0006	0.0009	-0.0007	0.0453	0.0003	0.0012
D12ATA63	p	0.0800	0.5899	0.7951	0.4424	0.8327	0.2499	0.5528	0.2750	0.6159	0.0000	0.3042	0.2475
	Fst	0.0005	-0.0014	-0.0027	-0.0010	-0.0022	-0.0002	-0.0013	-0.0029	-0.0009	0.0000	0.0002	-0.0016
D14S1434	p	0.2800	0.7698	0.8969	0.6330	0.9934	0.4044	0.8610	0.9129	0.7112	0.3697	0.3109	0.6619
	Fst	-0.0021	-0.0017	-0.0028	-0.0020	-0.0018	-0.0046	-0.0014	-0.0018	-0.0012	0.0091	-0.0006	0.0089
D17S1301	p	0.9883	0.8760	0.9322	0.9459	0.8962	0.9500	0.9279	0.6882	0.8211	0.0228	0.5618	0.0132
	Fst	-0.0011	-0.0010	-0.0034	-0.0023	-0.0023	-0.0048	-0.0017	-0.0026	-0.0016	-0.0023	-0.0013	0.0036
D18S853	p	0.6715	0.6366	0.9958	0.9975	0.9958	0.9592	0.9848	0.8364	0.9721	0.7250	0.8320	0.1048
	Fst	-0.0014	-0.0017	-0.0010	-0.0012	-0.0016	-0.0041	-0.0012	-0.0027	-0.0009	0.0067	-0.0014	-0.0018
D19S433	p	0.8628	0.9458	0.5923	0.7993	0.9169	0.9443	0.8934	0.9474	0.8157	0.0241	0.9549	0.7811
	Fst	-0.0013	0.0001	-0.0006	-0.0010	-0.0007	-0.0027	0.0017	-0.0017	0.0007	0.0168	0.0017	0.0009
D20S482	p	0.7504	0.3502	0.4665	0.6459	0.5503	0.6833	0.1149	0.6650	0.2172	0.0019	0.1113	0.2634
	Fst	0.0005	-0.0016	0.0024	0.0001	0.2733	0.0745	0.0014	0.0031	0.0012	0.0355	0.0033	0.0011
D22S1045	p	0.3057	0.8315	0.1498	0.3790	0.0000	0.0000	0.1453	0.1330	0.1630	0.0000	0.0366	0.2605

Supplementary Table S6. The Cavalli-Sforza and Edward's chord distance among 26 Chinese populations. LST: Lhasa-Tibetan; QHS: Qinghai-Salar; HBTJ: Hubei-Tujia; YNB: Yunnan-Bai; NXH: Ningxia-Han; AU: Kuqa-Uyghur; ILK: Ili-Kazakh; FJS: Fujian-She; IMM: Inner-Mongolia-Mongolian; NH: Northern-Han; GNH: Guanzhong-Han; YNY: Yunnan-Yi; HNL: Hainan-Li; GGH: Guangdong-Han; HNH: Hunan-Han; HDH: Huadong-Han; BJH: Beijing-Han; ZJH: Zhejiang-Han; IMR: Inner-Mongolia-Russian; SDH: Shandong-Han; LNH: Liaoning-Han; HNH-2: Henan-Han-2; GSYG: Gansu-Yugu; XJXO: Xinjiang-Xibe; HNH-1: Henan-Han-1; SCH: Sichuan-Han.

Populations	[01]	[02]	[03]	[04]	[05]	[06]	[07]	[08]	[09]	[10]	[11]	[12]	[13]
[01] XJXB													
[02] HNH-1	0.0084												
[03] SCH	0.0029	0.0076											
[04] LST	0.0060	0.0116	0.0055										
[05] QHS	0.0071	0.0116	0.0061	0.0092									
[06] HBTJ	0.0045	0.0091	0.0023	0.0070	0.0066								
[07] YNB	0.0044	0.0097	0.0028	0.0063	0.0076	0.0038							
[08] NXH	0.0037	0.0081	0.0019	0.0055	0.0064	0.0032	0.0037						
[09] AU	0.0039	0.0096	0.0031	0.0064	0.0065	0.0042	0.0049	0.0036					
[10] ILK	0.0048	0.0115	0.0058	0.0067	0.0080	0.0068	0.0065	0.0056	0.0036				
[11] FJS	0.0108	0.0154	0.0090	0.0122	0.0077	0.0099	0.0106	0.0102	0.0119	0.0147			
[12] IMM	0.0019	0.0073	0.0017	0.0053	0.0051	0.0027	0.0034	0.0019	0.0028	0.0041	0.0093		
[13] NH	0.0046	0.0102	0.0038	0.0076	0.0044	0.0048	0.0058	0.0045	0.0050	0.0081	0.0071	0.0036	
[14] GNH	0.0028	0.0077	0.0015	0.0050	0.0059	0.0026	0.0031	0.0020	0.0034	0.0053	0.0094	0.0014	0.0039
[15] YNY	0.0088	0.0144	0.0078	0.0115	0.0069	0.0081	0.0080	0.0086	0.0088	0.0113	0.0075	0.0076	0.0066
[16] HNL	0.0080	0.0122	0.0045	0.0104	0.0095	0.0059	0.0071	0.0061	0.0044	0.0094	0.0135	0.0055	0.0071
[17] GGH	0.0037	0.0084	0.0015	0.0067	0.0070	0.0023	0.0035	0.0026	0.0031	0.0068	0.0089	0.0023	0.0044
[18] HNH	0.0031	0.0079	0.0011	0.0060	0.0061	0.0021	0.0033	0.0024	0.0030	0.0061	0.0086	0.0017	0.0037
[19] HDH	0.0037	0.0089	0.0025	0.0067	0.0056	0.0035	0.0040	0.0029	0.0040	0.0070	0.0078	0.0024	0.0031
[20] BJH	0.0026	0.0074	0.0013	0.0052	0.0052	0.0023	0.0035	0.0018	0.0032	0.0055	0.0095	0.0011	0.0027
[21] ZJH	0.0037	0.0084	0.0025	0.0067	0.0069	0.0034	0.0045	0.0031	0.0040	0.0064	0.0100	0.0027	0.0042
[22] IMR	0.0077	0.0133	0.0074	0.0109	0.0057	0.0088	0.0089	0.0081	0.0076	0.0099	0.0087	0.0066	0.0049
[23] SDH	0.0022	0.0068	0.0011	0.0050	0.0055	0.0021	0.0031	0.0016	0.0032	0.0052	0.0085	0.0009	0.0033
[24] LNH	0.0033	0.0080	0.0021	0.0059	0.0058	0.0031	0.0039	0.0024	0.0039	0.0060	0.0092	0.0019	0.0040
[25] HNH-2	0.0022	0.0072	0.0011	0.0051	0.0051	0.0021	0.0030	0.0015	0.0031	0.0051	0.0085	0.0009	0.0032
[26] GSYG	0.0074	0.0130	0.0079	0.0088	0.0098	0.0085	0.0101	0.0076	0.0082	0.0074	0.0157	0.0062	0.0105

Supplementary Table S6. Continue

Populations	[14]	[15]	[16]	[17]	[18]	[19]	[20]	[21]	[22]	[23]	[24]	[25]	[26]
[01] XJXB													
[02] HNH-1													
[03] SCH													
[04] LST													
[05] QHS													
[06] HBTJ													
[07] YNB													
[08] NXH													
[09] AU													
[10] ILK													
[11] FJS													
[12] IMM													
[13] NH													
[14] GNH													
[15] YNY	0.0084												
[16] HNL	0.0059	0.0115											
[17] GGH	0.0021	0.0081	0.0036										
[18] HNH	0.0018	0.0074	0.0041	0.0011									
[19] HDH	0.0024	0.0058	0.0062	0.0027	0.0021								
[20] BJH	0.0014	0.0078	0.0044	0.0021	0.0014	0.0020							
[21] ZJH	0.0026	0.0084	0.0057	0.0028	0.0027	0.0033	0.0017						
[22] IMR	0.0072	0.0073	0.0109	0.0087	0.0074	0.0064	0.0066	0.0078					
[23] SDH	0.0011	0.0074	0.0052	0.0016	0.0011	0.0017	0.0008	0.0021	0.0068				
[24] LNH	0.0018	0.0085	0.0064	0.0027	0.0022	0.0027	0.0016	0.0030	0.0078	0.0015			
[25] HNH-2	0.0010	0.0071	0.0054	0.0017	0.0011	0.0017	0.0008	0.0023	0.0066	0.0004	0.0015		
[26] GSYG	0.0073	0.0148	0.0126	0.0085	0.0083	0.0082	0.0074	0.0086	0.0125	0.0070	0.0079	0.0069	

Supplementary Table S7. The Nei's genetic distance among 26 Chinese populations. LST: Lhasa-Tibetan; QHS: Qinghai-Salar; HBTJ: Hubei-Tujia; YNB: Yunnan-Bai; NXH: Ningxia-Han; AU: Kuqa-Uyghur; ILK: Ili-Kazakh; FJS: Fujian-She; IMM: Inner-Mongolia-Mongolian; NH: Northern-Han; GNH: Guanzhong-Han; YNY: Yunnan-Yi; HNL: Hainan-Li; GGH: Guangdong-Han; HNH: Hunan-Han; HDH: Huadong-Han; BJH: Beijing-Han; ZJH: Zhejiang-Han; IMR: Inner-Mongolia-Russian; SDH: Shandong-Han; LNH: Liaoning-Han; HNH-2: Henan-Han-2; GSYG: Gansu-Yugu; XJXO: Xinjiang-Xibe; HNH-1: Henan-Han-1; SCH: Sichuan-Han.

Populations	[01]	[02]	[03]	[04]	[05]	[06]	[07]	[08]	[09]	[10]	[11]	[12]	[13]
[01] XJXB													
[02] HNH-1	0.0376												
[03] SCH	0.0150	0.0320											
[04] LST	0.0318	0.0525	0.0284										
[05] QHS	0.0289	0.0477	0.0226	0.0360									
[06] HBTJ	0.0250	0.0416	0.0096	0.0279	0.0338								
[07] YNB	0.0177	0.0416	0.0117	0.0278	0.0270	0.0169							
[08] NXH	0.0170	0.0327	0.0073	0.0255	0.0269	0.0143	0.0116						
[09] AU	0.0205	0.0415	0.0088	0.0299	0.0295	0.0158	0.0157	0.0126					
[10] ILK	0.0170	0.0503	0.0270	0.0265	0.0359	0.0287	0.0229	0.0254	0.0199				
[11] FJS	0.0588	0.0761	0.0457	0.0589	0.0324	0.0518	0.0472	0.0524	0.0557	0.0701			
[12] IMM	0.0068	0.0319	0.0067	0.0225	0.0211	0.0128	0.0100	0.0060	0.0128	0.0166	0.0462		
[13] NH	0.0193	0.0421	0.0112	0.0357	0.0159	0.0179	0.0172	0.0148	0.0203	0.0327	0.0389	0.0116	
[14] GNH	0.0121	0.0325	0.0060	0.0233	0.0245	0.0118	0.0102	0.0052	0.0129	0.0225	0.0506	0.0041	0.0119
[15] YNY	0.0420	0.0710	0.0348	0.0583	0.0312	0.0409	0.0394	0.0409	0.0377	0.0431	0.0404	0.0338	0.0306
[16] HNL	0.0463	0.0598	0.0180	0.0570	0.0481	0.0267	0.0326	0.0265	0.0186	0.0585	0.0609	0.0319	0.0341
[17] GGH	0.0237	0.0427	0.0057	0.0391	0.0320	0.0104	0.0172	0.0126	0.0093	0.0358	0.0493	0.0137	0.0178
[18] HNH	0.0184	0.0356	0.0036	0.0334	0.0261	0.0093	0.0144	0.0103	0.0102	0.0312	0.0444	0.0095	0.0126
[19] HDH	0.0171	0.0368	0.0064	0.0318	0.0221	0.0121	0.0125	0.0085	0.0142	0.0300	0.0395	0.0076	0.0094
[20] BJH	0.0127	0.0310	0.0047	0.0254	0.0227	0.0111	0.0108	0.0068	0.0125	0.0247	0.0484	0.0050	0.0094
[21] ZJH	0.0165	0.0331	0.0069	0.0322	0.0271	0.0124	0.0153	0.0108	0.0145	0.0273	0.0485	0.0094	0.0142
[22] IMR	0.0303	0.0528	0.0257	0.0517	0.0194	0.0396	0.0297	0.0263	0.0319	0.0453	0.0429	0.0242	0.0135
[23] SDH	0.0114	0.0305	0.0041	0.0245	0.0227	0.0091	0.0101	0.0058	0.0120	0.0233	0.0457	0.0034	0.0098
[24] LNH	0.0116	0.0328	0.0065	0.0256	0.0229	0.0145	0.0116	0.0078	0.0150	0.0239	0.0485	0.0053	0.0127
[25] HNH-2	0.0105	0.0303	0.0042	0.0245	0.0223	0.0104	0.0114	0.0056	0.0114	0.0223	0.0470	0.0037	0.0090
[26] GSYG	0.0337	0.0501	0.0335	0.0332	0.0426	0.0341	0.0356	0.0266	0.0371	0.0346	0.0735	0.0251	0.0410

Supplementary Table S7. Continue

Populations	[14]	[15]	[16]	[17]	[18]	[19]	[20]	[21]	[22]	[23]	[24]	[25]	[26]
[01] XJXB													
[02] HNH-1													
[03] SCH													
[04] LST													
[05] QHS													
[06] HBTJ													
[07] YNB													
[08] NXH													
[09] AU													
[10] ILK													
[11] FJS													
[12] IMM													
[13] NH													
[14] GNH													
[15] YNY	0.0411												
[16] HNL	0.0275	0.0541											
[17] GGH	0.0133	0.0388	0.0120										
[18] HNH	0.0081	0.0362	0.0150	0.0040									
[19] HDH	0.0083	0.0294	0.0245	0.0086	0.0057								
[20] BJH	0.0046	0.0375	0.0245	0.0112	0.0057	0.0059							
[21] ZJH	0.0087	0.0364	0.0259	0.0115	0.0087	0.0098	0.0065						
[22] IMR	0.0260	0.0302	0.0500	0.0350	0.0288	0.0202	0.0232	0.0300					
[23] SDH	0.0037	0.0381	0.0254	0.0095	0.0048	0.0048	0.0018	0.0056	0.0254				
[24] LNH	0.0059	0.0405	0.0287	0.0132	0.0100	0.0097	0.0049	0.0097	0.0279	0.0046			
[25] HNH-2	0.0035	0.0360	0.0255	0.0099	0.0046	0.0049	0.0018	0.0067	0.0234	0.0013	0.0045		
[26] GSYG	0.0271	0.0669	0.0612	0.0364	0.0360	0.0304	0.0319	0.0355	0.0525	0.0276	0.0315	0.0284	

Supplementary Table S8. The Reynolds genetic distance among 26 Chinese populations. LST: Lhasa-Tibetan; QHS: Qinghai-Salar; HBTJ: Hubei-Tujia; YNB: Yunnan-Bai; NXH: Ningxia-Han; AU: Kuqa-Uyghur; ILK: Ili-Kazakh; FJS: Fujian-She; IMM: Inner-Mongolia-Mongolian; NH: Northern-Han; GNH: Guanzhong-Han; YNY: Yunnan-Yi; HNL: Hainan-Li; GGH: Guangdong-Han; HNH: Hunan-Han; HDH: Huadong-Han; BJH: Beijing-Han; ZJH: Zhejiang-Han; IMR: Inner-Mongolia-Russian; SDH: Shandong-Han; LNH: Liaoning-Han; HNH-2: Henan-Han-2; GSYG: Gansu-Yugu; XJXO: Xinjiang-Xibe; HNH-1: Henan-Han-1; SCH: Sichuan-Han.

Populations	[01]	[02]	[03]	[04]	[05]	[06]	[07]	[08]	[09]	[10]	[11]	[12]	[13]
[01] XJXB													
[02] HNH-1	0.0136												
[03] SCH	0.0056	0.0117											
[04] LST	0.0121	0.0196	0.0108										
[05] QHS	0.0105	0.0172	0.0083	0.0136									
[06] HBTJ	0.0093	0.0154	0.0036	0.0108	0.0125								
[07] YNB	0.0066	0.0154	0.0045	0.0107	0.0101	0.0065							
[08] NXH	0.0063	0.0120	0.0027	0.0098	0.0099	0.0054	0.0044						
[09] AU	0.0074	0.0147	0.0033	0.0113	0.0105	0.0060	0.0059	0.0047					
[10] ILK	0.0062	0.0180	0.0098	0.0101	0.0129	0.0106	0.0085	0.0093	0.0071				
[11] FJS	0.0224	0.0286	0.0177	0.0230	0.0128	0.0201	0.0184	0.0202	0.0212	0.0263			
[12] IMM	0.0025	0.0116	0.0025	0.0087	0.0077	0.0049	0.0038	0.0023	0.0046	0.0060	0.0179		
[13] NH	0.0070	0.0151	0.0041	0.0134	0.0058	0.0067	0.0064	0.0055	0.0072	0.0117	0.0152	0.0042	
[14] GNH	0.0045	0.0119	0.0023	0.0090	0.0090	0.0045	0.0039	0.0020	0.0048	0.0082	0.0195	0.0015	0.0044
[15] YNY	0.0152	0.0253	0.0128	0.0217	0.0114	0.0152	0.0146	0.0150	0.0135	0.0155	0.0158	0.0123	0.0111
[16] HNL	0.0164	0.0211	0.0066	0.0209	0.0170	0.0099	0.0120	0.0097	0.0066	0.0203	0.0231	0.0114	0.0121
[17] GGH	0.0088	0.0156	0.0022	0.0149	0.0118	0.0040	0.0065	0.0048	0.0036	0.0131	0.0192	0.0051	0.0066
[18] HNH	0.0067	0.0130	0.0013	0.0127	0.0096	0.0035	0.0054	0.0039	0.0038	0.0113	0.0173	0.0035	0.0046
[19] HDH	0.0064	0.0136	0.0024	0.0122	0.0082	0.0046	0.0048	0.0032	0.0053	0.0110	0.0155	0.0029	0.0036
[20] BJH	0.0047	0.0114	0.0018	0.0098	0.0084	0.0042	0.0041	0.0026	0.0047	0.0090	0.0187	0.0019	0.0035
[21] ZJH	0.0061	0.0120	0.0026	0.0122	0.0098	0.0047	0.0058	0.0040	0.0053	0.0099	0.0187	0.0035	0.0052
[22] IMR	0.0108	0.0187	0.0093	0.0191	0.0070	0.0145	0.0110	0.0096	0.0111	0.0159	0.0166	0.0087	0.0048
[23] SDH	0.0043	0.0113	0.0016	0.0094	0.0084	0.0035	0.0039	0.0022	0.0045	0.0085	0.0178	0.0013	0.0037
[24] LNH	0.0043	0.0120	0.0024	0.0098	0.0084	0.0055	0.0044	0.0029	0.0055	0.0087	0.0188	0.0020	0.0047
[25] HNH-2	0.0039	0.0112	0.0016	0.0094	0.0082	0.0040	0.0043	0.0021	0.0043	0.0082	0.0182	0.0014	0.0034
[26] GSYG	0.0132	0.0193	0.0132	0.0132	0.0165	0.0135	0.0140	0.0106	0.0145	0.0136	0.0288	0.0100	0.0159

Supplementary Table S8. Continue

Populations	[14]	[15]	[16]	[17]	[18]	[19]	[20]	[21]	[22]	[23]	[24]	[25]	[26]
[01] XJXB													
[02] HNH-1													
[03] SCH													
[04] LST													
[05] QHS													
[06] HBTJ													
[07] YNB													
[08] NXH													
[09] AU													
[10] ILK													
[11] FJS													
[12] IMM													
[13] NH													
[14] GNH													
[15] YNY	0.0150												
[16] HNL	0.0100	0.0192											
[17] GGH	0.0050	0.0143	0.0045										
[18] HNH	0.0030	0.0132	0.0055	0.0015									
[19] HDH	0.0031	0.0109	0.0090	0.0033	0.0022								
[20] BJH	0.0017	0.0137	0.0089	0.0042	0.0021	0.0022							
[21] ZJH	0.0032	0.0132	0.0093	0.0043	0.0032	0.0037	0.0024						
[22] IMR	0.0095	0.0109	0.0173	0.0127	0.0104	0.0075	0.0084	0.0107					
[23] SDH	0.0014	0.0140	0.0093	0.0036	0.0018	0.0018	0.0007	0.0021	0.0093				
[24] LNH	0.0022	0.0148	0.0104	0.0050	0.0037	0.0036	0.0019	0.0036	0.0101	0.0017			
[25] HNH-2	0.0013	0.0132	0.0093	0.0038	0.0017	0.0018	0.0007	0.0025	0.0085	0.0005	0.0017		
[26] GSYG	0.0108	0.0253	0.0231	0.0143	0.0141	0.0120	0.0126	0.0139	0.0200	0.0110	0.0124	0.0113	

Supplementary Table S9. The Nei's genetic distance among 13 Chinese Han populations.

Populations	[01]	[02]	[03]	[04]	[05]	[06]	[07]	[08]	[09]	[10]	[11]	[12]	[13]
[01] Henan-Han-1													
[02] Sichuan-Han	0.03198												
[03] Ningxia-Han	0.03267	0.00725											
[04] Northern-Han	0.04205	0.01119	0.01481										
[05] Guanzhong-Han	0.03248	0.00603	0.00518	0.01191									
[06] Guangdong-Han	0.04266	0.00571	0.01264	0.01781	0.01325								
[07] Hunan-Han	0.03556	0.00358	0.01031	0.01256	0.00811	0.00401							
[08] Huadong-Han	0.03681	0.00643	0.00854	0.00941	0.00828	0.00860	0.00571						
[09] Beijing-Han	0.03098	0.00468	0.00682	0.00942	0.00456	0.01121	0.00567	0.00590					
[10] Zhejiang-Han	0.03307	0.00686	0.01081	0.01416	0.00869	0.01148	0.00868	0.00985	0.00651				
[11] Shandong-Han	0.03055	0.00414	0.00576	0.00985	0.00368	0.00952	0.00476	0.00478	0.00183	0.00564			
[12] Liaoning-Han	0.03282	0.00650	0.00782	0.01273	0.00587	0.01319	0.01001	0.00966	0.00494	0.00967	0.00461		
[13] Henan-Han-2	0.03033	0.00423	0.00555	0.00901	0.00346	0.00993	0.00457	0.00486	0.00176	0.00668	0.00128	0.00453	
